# Antibiotic resistance gene sharing networks and the effect of dietary nutritional content on the canine and feline gut resistome

**DOI:** 10.1186/s42523-020-0022-2

**Published:** 2020-02-07

**Authors:** Younjung Kim, Marcus H. Y. Leung, Wendy Kwok, Guillaume Fournié, Jun Li, Patrick K. H. Lee, Dirk U. Pfeiffer

**Affiliations:** 1 Department of Infectious Diseases and Public Health, Jockey Club College of Veterinary Medicine and Life Sciences, City University of Hong Kong, Hong Kong, China; 2 School of Energy and Environment, City University of Hong Kong, Hong Kong, China; 3 Department of Pathobiology and Population Sciences, Royal Veterinary College, London, UK; 4 School of Data Science, City University of Hong Kong, Hong Kong, China

**Keywords:** Cat microbiome, Diet, Dietary protein content, Dog microbiome, Gut microbiome, Horizontal gene transfer, Metagenomics, Network analysis

## Abstract

**Background:**

As one of the most densely populated microbial communities on Earth, the gut microbiota serves as an important reservoir of antibiotic resistance genes (ARGs), referred to as the gut resistome. Here, we investigated the association of dietary nutritional content with gut ARG diversity and composition, using publicly available shotgun metagenomic sequence data generated from canine and feline fecal samples. Also, based on network theory, we explored ARG-sharing patterns between gut bacterial genera by identifying the linkage structure between metagenomic assemblies and their functional genes obtained from the same data.

**Results:**

In both canine and feline gut microbiota, an increase in protein and a reduction in carbohydrate in the diet were associated with increased ARG diversity. ARG diversity of the canine gut microbiota also increased, but less strongly, after a reduction in protein and an increase in carbohydrate in the diet. The association between ARG and taxonomic composition suggests that diet-induced changes in the gut microbiota may be responsible for changes in ARG composition, supporting the links between protein metabolism and antibiotic resistance in gut microbes. In the analysis of the ARG-sharing patterns, 22 ARGs were shared among 46 genera in the canine gut microbiota, and 11 ARGs among 28 genera in the feline gut microbiota. Of these ARGs, the tetracycline resistance gene *tet*(W) was shared among the largest number of genera, predominantly among *Firmicutes* genera. *Bifidobacterium*, a genus extensively used in the fermentation of dairy products and as probiotics, shared *tet*(W) with a wide variety of other genera. Finally, genera from the same phylum were more likely to share ARGs than with those from different phyla.

**Conclusions:**

Our findings show that dietary nutritional content, especially protein content, is associated with the gut resistome and suggest future research to explore the impact of dietary intervention on the development of antibiotic resistance in clinically-relevant gut microbes. Our network analysis also reveals that the genetic composition of bacteria acts as an important barrier to the horizontal transfer of ARGs. By capturing the underlying gene-sharing relationships between different bacterial taxa from metagenomes, our network approach improves our understanding of horizontal gene transfer dynamics.

## Background

The widespread use of antibiotics in human medicine, veterinary medicine, and agriculture has created unremitting selection pressure for antibiotic resistance since antibiotics were first introduced in the 1940s [1]. Although antibiotic resistance has become a global health concern over the past few decades, genes conferring resistance to antibiotics have long preceded antibiotic discovery and usage, offering survival advantages to host microbes through the various metabolic and regulatory roles they play [1]. The gut microbiota is one of the most densely populated microbial communities on Earth [2, 3] and therefore serves as an important reservoir of antibiotic resistance genes (ARGs), referred to as the gut resistome [4]. The intestinal tract is colonized by commensals as well as opportunistic pathogens, and is constantly exposed to pathogenic and non-pathogenic microbes via food and water. These microbes have ample opportunity to interact closely with each other. As a result, the gut provides an ideal environment for the horizontal transfer of ARGs between different members of the gut microbiota [4, 5].

In this study, we aimed to examine two different aspects of the gut microbiota, using publicly available shotgun metagenomic sequence data generated from canine and feline fecal samples. The first objective was to assess whether dietary nutritional content was associated with gut ARG diversity and composition by comparing these across different diet groups. Diet is one of the most influential factors shaping the gut microbiota [6–10]. However, most studies exploring the impact of diet on the gut microbiota have used amplicon sequence data and therefore focused on the taxonomic profile of gut microbes. Some have expanded their scope to the functional profile using shotgun sequence data, but only a few have explored the influence of diet on the gut resistome [11]. Given the inextricable link between microbes and ARGs, we hypothesize that diet-induced alteration in the gut microbiota changes gut ARG diversity and composition, that is, the antibiotic resistance potential of the gut microbiota.

The second objective was to understand ARG-sharing relationships between gut bacterial genera by constructing ARG-sharing networks between genera, identifying genera that may play a key role in the horizontal transfer of ARGs, and assessing the extent to which ARG sharing between genera is constrained by bacterial taxonomic classification. We defined ARG sharing as the presence of a given ARG in different bacterial taxa. The recognition that horizontal gene transfer (HGT) plays a significant role in microbial evolution has encouraged us to consider a microbial community as a network of actors sharing genes. Recent studies have explored gene-sharing relationships between microbial genomes by applying network approaches to whole-genome sequence data [12–15]. However, while these studies have expanded our understanding of microbial evolution via HGT, they are limited in their capacity to describe the complex dynamics of HGT occurring in a particular microbial community, because they used bacterial genomes isolated from various microbial communities. Here, we present a network approach that captures the underlying network structure between metagenomic assemblies and their functional genes originating from a particular microbial community.

## Results

### The dietary effect on the gut resistome

**Fig. 1:**
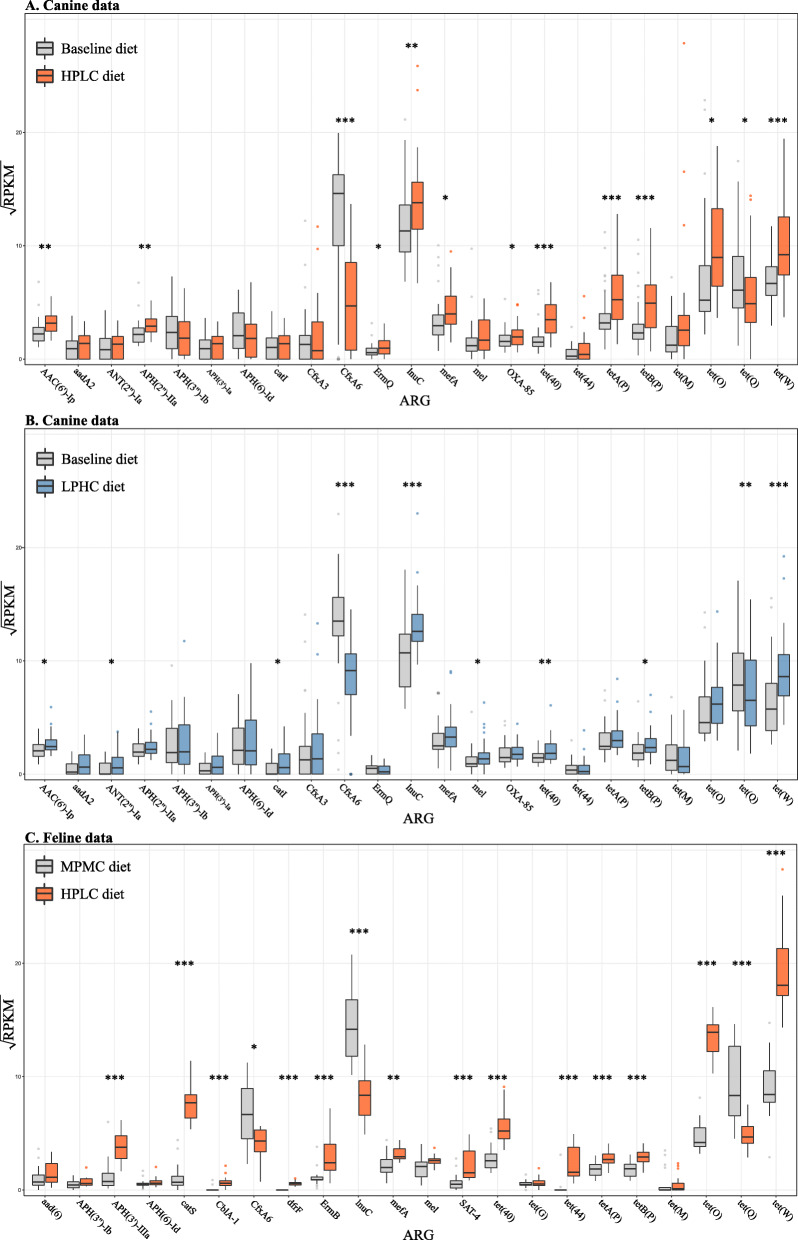
Boxplots showing the square root transformed ARG abundance in the canine and feline gut microbiota. Reads per kilobase of transcript per million mapped reads (RPKM) was used as the measure of ARG abundance. Boxplots show the abundance of a given ARG before and after intervention with HPLC (**a**) and LPHC (**b**) diets in the canine data, respectively, and between different MPMC and HPLC diet groups in the feline data (**c**). Non-parametric statistical methods were used. For the canine data, the Wilcoxon signed-rank test was used since samples collected from the same animals comprised different diet groups. For the feline data, the Wilcoxon rank-sum test was used (*: *p* < 0.05, **: *p* < 0.01, ***: *p* < 0.001)

**Fig. 2:**
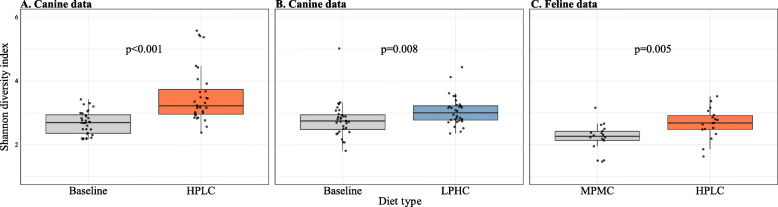
The Shannon diversity index before and after intervention with HPLC (**a**) and LPHC (**b**) diets in the canine data, and between different MPMC and HPLC diet groups in the feline data (**c**). Non-parametric statistical methods were used. For the canine data, the Wilcoxon signed-rank test was used since samples collected from the same animals comprised different diet groups. For the feline data, the Wilcoxon rank-sum test was used

A total of 23 ARGs were identified in ≥50% of the samples in both canine and feline data, with tetracycline and aminoglycoside resistance genes being the most frequent ARGs (Fig. 1) (see Additional file 1: Table S1 for the statistics of de novo assembly). The abundance of a given ARG tended to respond to dietary intervention similarly in both canine and feline data. For example, dogs with the High-Protein/Low-Carbohydrate (HPLC) diet tended to have a higher abundance of *tet*(W), *tet*(O), *tet* (44) (tetracycline resistance genes), *mefA* and *mel* (macrolide resistance genes), but a lower abundance of *CfxA6* (a beta-lactam antibiotic resistance gene), compared with dogs with the baseline diet (Figs. 1a). The abundance of these ARGs showed a similar pattern between HPLC-fed kittens and Moderate-Protein/Moderate-Carbohydrate (MPMC)-fed kittens (Fig. 1c). Dietary nutritional content also influenced overall diversity of ARGs in both canine and feline gut data. In dogs, changes of diet from the baseline to HPLC and Low-Protein/High-Carbohydrate (LPHC) diets were both associated with a significant increase in the Shannon diversity index of ARGs (*p* < 0.001 and *p* = 0.008, respectively, Wilcoxon signed-rank test) (Fig. 2a–b). This increase was more pronounced with the HPLC diet than with the LPHC diet; the mean Shannon diversity index of ARGs increased by 31.5% with the HPLC diet, whereas it increased by approximately 10.2% with the LPHC diet. This resulted in the mean Shannon diversity index of ARGs being 15.7% higher in HPLC- than LPHC-fed dogs (*p* = 0.023, Wilcoxon rank-sum test). Likewise, the mean Shannon diversity index of ARGs was 19.8% higher in HPLC-fed kittens than MPMC-fed kittens (*p* = 0.005, Wilcoxon rank-sum test) (Fig. 2c). As for taxonomic diversity, HPLC- and LPHC-fed dogs had 11.2 and 14.8% higher mean Shannon diversity index of bacterial genera than dogs with the baseline diet (all *p* < 0.001, Wilcoxon signed-rank test). Also, the mean Shannon diversity index of bacterial genera was 26.2% higher in HPLC-fed kittens than MPMC-fed kittens (*p* < 0.001, Wilcoxon rank-sum test).

**Fig. 3:**
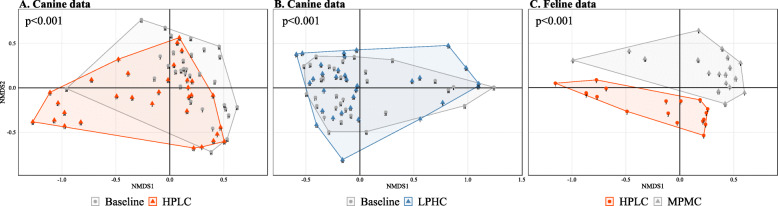
ARG composition before and after intervention with HPLC **(a**, stress = 0.15**)** and LPHC **(b**, stress = 0.16**)** diets in the canine data, and between different HPLC and MPMC diet groups in the feline data **(c**, stress = 0.10**)**. In both data, there were statistically significant associations between diet type and ARG composition (all *p* < 0.001, permutational multivariate analysis of variance test)

**Fig. 4:**
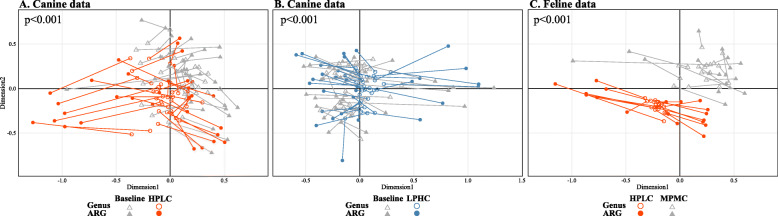
Procrustes analysis of the association between ARG and taxonomic composition. Samples from the same animals are connected by a line, with hollow and filled points representing samples positioned by bacterial and ARG composition, respectively. In the canine data, red and blue circles represent samples with HPLC (**a**) and LPHC (**b**) diets, respectively, whereas grey triangles represent the baseline diet (**a** and **b**). In the feline data (**c**), red circles represent samples with HPLC diet, and grey triangles represent samples with MPMC diet. Taxonomic composition was assessed at the genus level. In both canine (**a** and **b**) and feline (**c**) data, there were statistically significant associations between ARG and taxonomic composition (all *p* < 0.001, procrustean randomization test), suggesting that gut bacteria and ARGs have similar clustering patterns

When ARG composition was assessed between the diet groups based on Bray-Curtis dissimilarity values, there was a statistically significant association between ARG composition and diet type in both canine and feline data (all *p* < 0.001, permutational multivariate analysis of variance (PERMANOVA) test). In particular, HPLC-fed dogs showed a more distinct separation from those with a baseline diet than LPHC-fed dogs, as visualized in nonmetric multidimensional scaling (NMDS) ordinations (Fig. 3a–b). Also, there was a clear separation between HPLC-fed kittens and MPMC-fed kittens in the feline data (Fig. 3c). Procrustes analysis showed a statistically significant association between ARG and taxonomic composition in both canine and feline data (Fig. 4, all *p* < 0.001, procrustean randomization test), suggesting that samples with a similar taxonomic composition were more likely to show similar patterns of ARG composition than samples exhibiting different taxonomic composition.

### Antibiotic resistance gene-sharing relationships between gut bacterial genera

**Fig. 5:**
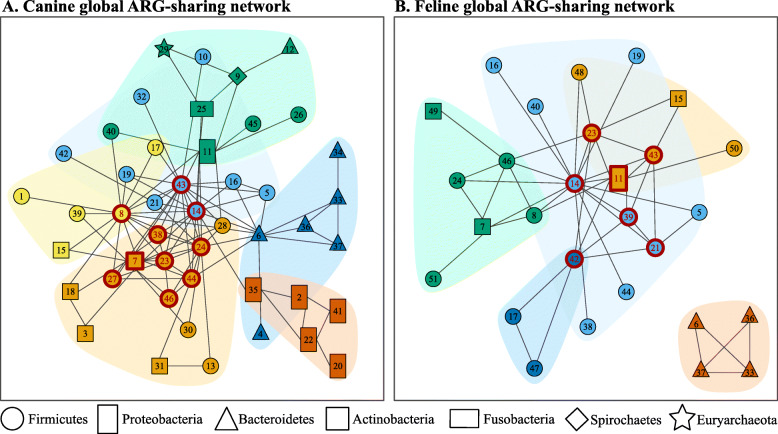
The global ARG-sharing network of the canine (**a**) and feline (**b**) gut microbiota. Nodes represent genera, with their shapes and colors representing phylum and network community memberships, respectively. Nodes with the same shape represent genera from the same phylum. Nodes with the same color represent genera classified into the same network community, based on the network structure; bacterial genera in the same network community shared ARGs more frequently among themselves than with genera belonging to other network communities. Two genera were connected by an edge if their contigs shared ≥1 ARG in ≥1 sample. Genera were classified as central (red border and label) and peripheral (black border and label) genera based on their structural equivalence. Node labels are IDs of genera (Table 1)

**Fig. 6:**
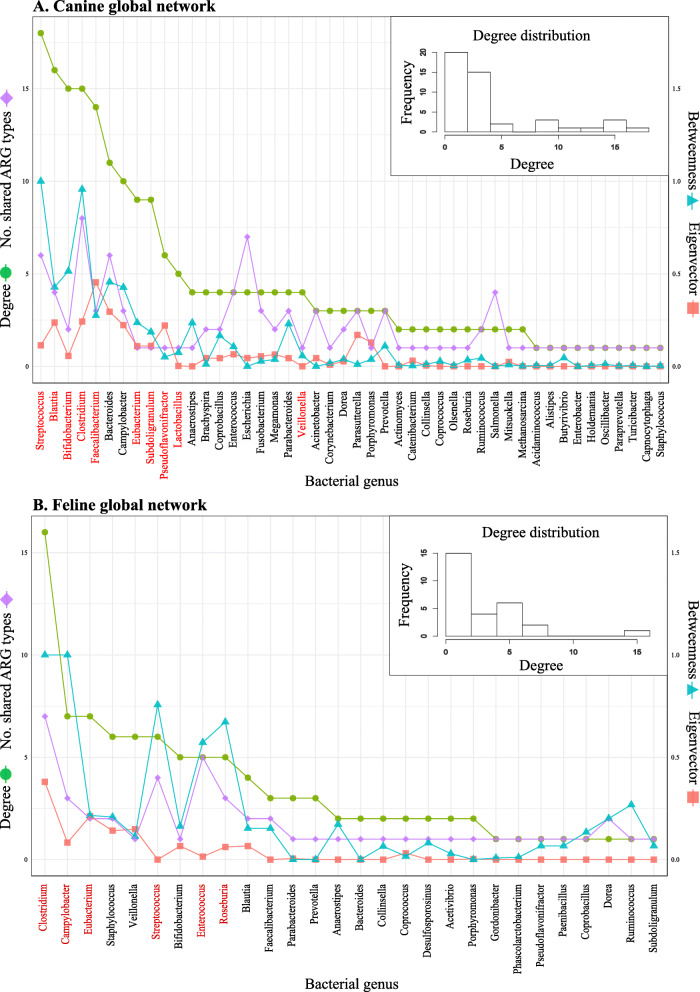
Centrality and the number of shared ARG types in the global ARG-sharing network of the canine (**a**) and feline (**b**) gut microbiota. The number of shared ARG types represents the number of ARG types a given genus shared with other genera. Genera are classified as central (red label) and peripheral (black label) genera based their structural equivalence. The histogram represents the degree distribution of each ARG-sharing network

We constructed two different types of ARG-sharing network: (i) global networks including all ARGs identified, and (ii) ARG-specific networks for which only one specific ARG was accounted for. A total of 46 and 28 bacterial genera were connected through the sharing of 22 and 11 ARGs in the canine and feline global networks, respectively (Fig. 5) (see Table 1 for bacterial genera and Table 2 for shared ARGs). Twenty-three genera and seven ARGs appeared in both networks. Tetracycline resistance genes were most commonly shared in both networks, followed by macrolide and aminoglycoside resistance genes, with *tet*(W) being detected in at least two genera in 93.8% (*n* = 60/64) of dogs and 75.0% (*n* = 9/12) of cats (Table 2). While a substantial majority of the genera were connected to a relatively small number of other genera, some were connected to a remarkably large number of other genera (Fig. 6). In particular, *Streptococcus* and *Clostridium* shared ARGs with the largest number of other genera in the canine and feline networks, respectively (Fig. 6). Although centrality measures (i.e., degree, eigenvector, and betweenness) tended to be positively correlated with one another, none of them was correlated with the number of ARG types shared by each genus (Additional file 2: Table S2). For example, *Bifidobacterium* shared only one ARG type in the feline network and two in the canine network, but with a large number of other genera (Fig. 6).

**Table 1:** List of bacterial genera in the canine and feline global networks

ID	Genus	ID	Genus	ID	Genus
1	*Acidaminococcus*	18	*Corynebacterium*	35	*Parasutterella*
2	*Acinetobacter*	19	*Dorea*	36	*Porphyromonas*
3	*Actinomyces*	20	*Enterobacter*	37	*Prevotella*
4	*Alistipes*	21	*Enterococcus*	38	*Pseudoflavonifractor*
5	*Anaerostipes*	22	*Escherichia*	39	*Roseburia*
6	*Bacteroides*	23	*Eubacterium*	40	*Ruminococcus*
7	*Bifidobacterium*	24	*Faecalibacterium*	41	*Salmonella*
8	*Blautia*	25	*Fusobacterium*	42	*Staphylococcus*
9	*Brachyspira*	26	*Holdemania*	43	*Streptococcus*
10	*Butyrivibrio*	27	*Lactobacillus*	44	*Subdoligranulum*
11	*Campylobacter*	28	*Megamonas*	45	*Turicibacter*
12	*Capnocytophaga*	29	*Methanosarcina*	46	*Veillonella*
13	*Catenibacterium*	30	*Mitsuokella*	47	*Acetivibrio*
14	*Clostridium*	31	*Olsenella*	48	*Desulfosporosinus*
15	*Collinsella*	32	*Oscillibacter*	49	*Gordonibacter*
16	*Coprobacillus*	33	*Parabacteroides*	50	*Paenibacillus*
17	*Coprococcus*	34	*Paraprevotella*	51	*Phascolarctobacterium*

The IDs of genera correspond to node labels in Fig. 5

**Table 2:** The frequency of ARG sharing among contigs

ARG type	Canine data (*n* = 64)				Feline data (*n* = 12)			
	No. animals ^a^	No. genera ^b^	No. phyla ^c^	No. sharing ^d^	No. animals ^a^	No. genera ^b^	No. phyla ^c^	No. sharing ^d^
*tet*(W)	60	21	4	213	9	12	2	19
*mefA*	39	8	2	75	2	3	1	4
*tet*(O)	37	4	2	46	5	5	2	18
*tet*(Q)	29	7	2	73	7	4	1	12
*mel*	24	9	3	51	4	4	1	5
*lnuC*	13	8	4	20	3	4	2	5
*OXA-85*	11	4	4	16	–	–		–
*APH(3″)-Ib*	9	3	1	15	–	–		–
*tet*(M)	6	2	1	7	–	–		–
APH(6)-Id	5	4	1	5	–	–		–
*tet*(44)	4	3	1	9	4	4	1	5
*CfxA3*	4	3	1	4	–	–		–
*CfxA6*	3	3	1	4	–	–		–
*tet*(32)	2	3	1	4	–	–		–
*mdtM*	2	2	1	2	–	–		–
*lnuA*	2	2	1	3	–	–		–
*bacA*	2	2	1	4	–	–		–
*cmeB*	1	2	2	1	–	–		–
*AAC(6′)-Ip*	1	2	2	1	–	–		–
*aadA2*	1	2	1	2	–	–		–
*mdtO*	1	2	1	2	–	–		–
*mdtP*	1	2	1	1	–	–		–
*ermB*	–				3	2	1	7
*aad(6)*	–				4	7	2	10
*catS*	–				3	3	1	5
*SAT-4*	–				1	2	1	1

^a^ The number of animals in which a given ARG was shared by contigs belonging to different bacterial genera at least once

^b^ The number of bacterial genera that shared a given ARG with other bacterial genera

^c^ The number of bacterial phyla that shared a given ARG with other bacterial genera

^d^ The frequency that a given ARG was found in two contigs annotated to different bacterial genera across the animals of a given animal species

In both canine and feline global networks, bacterial genera were more likely to share ARGs with other genera from the same phylum than genera belonging to different phyla, although this pattern was not statistically significant in the feline network. The odds of sharing ≥1 ARG with genera from the same phylum were 4.0 times as high in the canine network (*p* < 0.001, Quadratic Approximation Procedure (QAP) permutation test), and 2.3 times as high in the feline network (*p* = 0.164, QAP permutation test), than the odds of sharing ≥1 ARG with genera belonging to different phyla (Additional file 3: Table S3). The fast greedy modularity optimisation algorithm partitioned the canine and feline global networks into six and five network communities, respectively, which maximized the extent to which ARG sharing occurs within communities (Fig. 5 and Table 1) [16]. The network partitions were associated with phylum membership; genera from the same phylum were more likely to be classified into the same network community than those from different phyla in both canine (odds ratio = 4.6, *p* < 0.001, QAP permutation test) and feline (odds ratio = 3.9, *p* < 0.001, QAP permutation test) networks (Additional file 3: Table S3). The canine and feline global networks were also partitioned based on structural equivalence between genera. For example, two genera were considered structurally equivalent if they were connected to the same set of other genera through ARG sharing [17]. In both global networks, genera were classified as one of two structurally equivalent groups, central and peripheral genera, with central genera having higher centrality measures than peripheral genera (Figs. 5 and 6, and Table 1). *Streptococcus, Clostridium,* and *Eubacterium* were classified as central genera in both networks. Furthermore, while over 75% of all possible connections between central genera were present, peripheral genera were weakly connected to other peripheral and central genera (Additional file 4: Table S4).

The ARG-specific networks are presented in Figs. S1–2 and Tables S5–6 (Additional file 5). The canine and feline *tet*(W) networks were the largest, consisting of 21 and 12 bacterial genera belonging to four and two different phyla, respectively (Table 2). While *Bifidobacterium* had the highest centrality measures in the canine *tet*(W) network, *Clostridium* and *Veillonella* had the highest centrality measures in the feline *tet*(W) network, followed by *Bifidobacterium*. Macrolide resistance genes (e.g., *mefA* and *mel*) and other tetracycline resistance genes, such as *tet*(O), *tet*(Q), and *tet*(44), formed relatively large canine and feline ARG-specific networks (Additional file 5: Figure S1–2). However, most of these ARGs were shared predominantly within a particular phylum. For example, in both canine and feline ARG-specific networks, *tet*(O), *tet*(44), *mefA*, and *mel* were shared mostly or exclusively among *Firmicutes* genera, and *tet*(Q) among *Bacteroidetes* genera (Additional file 5: Tables S7–8).

## Discussion

It is essential to identify factors shaping the gut resistome and understand the dynamics of ARG transfer between gut bacteria to fully appreciate the antibiotic resistance potential of the gut microbiota. Our study shows that dietary nutritional content has implications for the gut microbiota as the reservoir of ARGs. The most intriguing finding is that the HPLC diet increased ARG diversity and altered ARG composition. These changes were likely to be driven by the changes in the gut microbiota, as suggested by the association between ARG and taxonomic composition in our study. The gut resistome depends on the gut microbiota because ARGs are generally integrated into bacterial genomes, except when they are mobilized for HGT. However, it is unclear why the HPLC diet particularly increased ARG diversity in both canine and feline data. Our study showed that both taxonomic and ARG diversity increased with the HPLC diet. However, if bacteria that increased in abundance with the HPLC diet tended to harbor fewer ARGs, depending on the initial status of the gut resistome, this could have decreased ARG diversity, contrary to our observations in the present study. Additionally, after dietary intervention, the increase in ARG diversity was higher with the HPLC than LPHC diet, despite a larger increase in taxonomic diversity with the LPHC than HPLC diet. This suggests that the overall increase in taxonomic diversity alone might not explain the overall increase in ARG diversity.

One possible explanation may be that genes for protein metabolism and antibiotic resistance have been co-selected in certain gut bacteria [18]. In support of this, we note that animal protein is the primary source of protein in most commercial pet foods, as in those used in both the canine and feline studies [2, 3]. Antibiotics are used extensively in food animals, leading to increasing levels of antibiotic-resistant bacteria and antibiotic residues in animal products [19–21]. Having been exposed to animal protein under this circumstance, bacteria adapted to protein fermentation could have had more opportunities to develop antibiotic resistance than those adapted to the fermentation of other macronutrients. Therefore, once genes for protein metabolism and antibiotic resistance are co-selected [18], a protein-rich diet could increase the abundance of bacteria promoting protein fermentation and, consequently, the abundance of ARGs carried by these bacteria, in the gut.

However, these findings should be interpreted with care. Even though overall ARG diversity increased with the HPLC diet, this was not always the case when the individual ARG abundances were compared between the diet groups. For example, the abundance of some ARGs such as the lincosamide resistance gene *lnuC* and the beta-lactamase resistance gene *CfxA6* decreased with the HPLC diet. Additionally, in contradiction to our hypothesis, overall ARG diversity also increased with the LPHC diet in the canine data, although the magnitude of the increase was lower than with the HPLC diet. These observations could be explained by the fact that the diets differed not only in protein content but also in their content of other macronutrients and the source of ingredients. In particular, the increase in ARG diversity with the LPHC diet was likely to be caused by differences other than protein content, because protein content of the LPHC diet was similar to the baseline diet, whereas protein content in the HPLC diet was almost twice as high as that of the baseline diet [2].

Some of the ARGs whose abundance was altered with dietary intervention also deserve special attention because they are known to confer resistance to antibiotics used frequently in primary care small animal veterinary practices (e.g., *CfxA6* for beta-lactam antibiotics) or to those classified as critically important by the World Health Organization (e.g., *ermB*, *mefA*, and *mel* for macrolides) [22, 23]. These findings suggest future research to explore the clinical implications of dietary intervention in dogs and cats. In particular, it should be noted that dietary intervention forms the mainstay of chronic enteropathy management in these animals, and diets recommended for chronic enteropathies have different nutritional content from standard diets because they are generally hydrolyzed, highly digestible, and moderately fat-restricted [24]. Therefore, future research could investigate whether the dietary management of chronic enteropathies influences the antibiotic potential of the gut microbiota and whether such influences are linked to the development of antibiotic resistance in clinically-relevant gut microbes. Such research will be of particular importance because antibiotics are used in the second-line treatment of chronic enteropathies, following dietary management.

Our study also investigated the sharing of ARGs between bacterial taxa by identifying the linkage structure between metagenomic assemblies and their functional genes obtained from canine and feline fecal samples. Although gene sharing does not necessarily provide direct evidence for HGT, network approaches can provide new insights into microbial evolution because HGT inevitably creates networks of microbes over a wide range of evolutionary distances [12, 25]. Several studies have employed network approaches to understand the gene-sharing relationships between microbial genomes [12–14, 26]. The gene-sharing networks of these studies were constructed from the genomes of microbes isolated from different origins and are therefore useful in providing information on the cumulative impact of HGT over a long evolutionary timescale. However, the findings of these studies were inherently limited to the selected genomes and might not adequately explain the dynamics of HGT occurring in a particular ecological niche, especially those considered hotspots of HGT (e.g., the gut). In this regard, our network approach should make important contributions to the field of microbial ecology, because it allows us to study the gene-sharing relationships between bacterial taxa based on metagenomes originating from a particular ecological niche. Here, we focused on ARGs, but our approach could be extended to all genes to provide broader insights into functional relationships between co-existing microorganisms.

Our networks show the extensive sharing of ARGs between a wide variety of genera in the canine and feline gut microbiota. The findings that genera from the same phylum tended to share ARGs and be classified into the same network community suggest that differences in the genetic composition of bacteria may limit the transfer and survival of ARGs in the new host genome. In particular, most ARGs tended to be shared exclusively by specific phyla. For example, *tet*(Q) was predominantly shared between *Bacteroidetes* genera in our study. *tet*(Q) has been associated with plasmids and conjugative transposons generally found in *Bacteroides* and close relatives, such as *Prevotella* and *Porphyromonas* (27–30). If these transmissible elements have been adapted to *Bacteroidetes* bacteria, they might have limited capacity to transfer genes to non-*Bacteroidetes* bacteria.

However, it should also be noted that certain ARGs, such as *tet*(W) and *lnuC*, were shared extensively between different phyla, suggesting that transmissible elements involved in the transfer of these ARGs may have broad host ranges. In particular, *tet*(W) networks comprised the largest ARG-specific networks, consistent with the fact that *tet*(W) is one of the most prevalent tetracycline resistance genes in mammalian gut bacteria [27]. *Bifidobacterium* had the highest centrality in both canine and feline *tet*(W) networks, suggesting that this genus has the potential to modulate the HGT dynamics of *tet*(W). Its high centrality could be explained by the flanking of *tet*(W) by transposase genes in *Bifidobacterium* [28]. Transposase is an enzyme that catalyzes the movement of DNA fragments within and between bacterial genomes [28]. Thus, its presence could have facilitated the horizontal transfer of *tet*(W) from *Bifidobacterium* to other bacteria in the canine and feline gut microbiota. Considering the widespread use of *Bifidobacterium* in the fermentation of dairy products and as probiotics [29, 30], our finding suggests that the presence and horizontal transfer of *tet*(W) should be closely monitored when *Bifidobacterium* is used in food products.

Our study has some limitations. First, although MyTaxa, a homology-based taxonomy classifier used to annotate contigs to bacterial genera and phyla, has relatively high accuracy at the phylum and genus levels and is considered to be superior to other annotation tools [31], it is still possible that some contigs were incorrectly annotated, leading to classification bias in the study results. If such misclassifications occurred and were biased towards specific bacterial taxa, it could result in overestimation of the influence of these bacteria in the networks. Second, our network approach is dependent on the assembly of short reads. Thus, low-abundance bacteria and ARGs might not have been included in the networks if their sequencing depths were insufficient to be assembled into contigs [32]. Additionally, the canine and feline networks were constructed with different numbers of samples. Therefore, different numbers of genera in the canine and feline networks might have been caused partly by different sequencing depths and sample sizes, in addition to inter-species differences in the gut microbiota. Third, we used 100% pairwise BLASTN sequence identity as the threshold for the most recent HGT events. However, edges in the networks might not necessarily represent HGT events that occurred at the same molecular timescale because different ARGs could have different mutation rates. Thus, accounting for ARG-specific mutation rates (should such information become available) would allow more reliable construction of ARG-sharing networks.

## Conclusions

Our study shows that dietary nutritional content alters the antibiotic resistance potential of the gut microbiota, supporting the hypothesis that there are intrinsic links between protein metabolism and antibiotic resistance. Future research should investigate whether such alteration in the gut resistome is indeed linked to the development of antibiotic resistance in clinically-relevant gut microbes. Our network approach shows the extensive sharing of ARGs across a wide range of canine and feline gut bacteria, suggesting that the gut microbiota serves as an important ARG reservoir and HGT hotspot. The modular network structure reflects the barriers to ARG spreading between bacterial genera, with phylum membership playing a significant role.

## Methods

### Study population and metagenomic data

We analyzed publicly available shotgun metagenomic sequence data generated by two previous studies [2, 3]. These studies assessed the impact of dietary nutritional content on the canine and feline gut microbiota, with a particular focus on the overall taxonomic and functional profiles of gut microbes. Briefly, 128 fecal samples were collected from 64 dogs, and 36 fecal samples from 12 cats, and their sequence data were used in our study as canine and feline data, respectively. In the canine study, 64 dogs received a baseline diet for the first 4 weeks. They were then equally split into two groups, each receiving for the next 4 weeks one of two intervention diets that mainly differed in protein and carbohydrate content: HPLC or LPHC. On a dry matter basis, protein content was highest in the HPLC diet (53.9%). The baseline and LPHC diet had relatively similar protein content at 29.9 and 27.3%, respectively [2]. Fecal samples were collected once before and once after dietary intervention. In the feline study, 12 kittens were split into two diet groups of equal size: HPLC or MPMC. On a dry matter basis, protein content was 52.9% in the HPLC diet and 34.3% in the MPMC diet [3]. They were housed with their mothers until 8 weeks of age and fed the same diets as their mothers after weaning. Three fecal samples were collected from each kitten at approximately 8, 12, and 16 weeks of age. The information on study design and dietary nutritional content is provided in detail in the previous studies [2, 3].

### Taxonomic and antibiotic resistance gene annotation

After removing paired-end reads with low-quality bases (quality scores < 20), reads < 30 bases, and PCR duplicates from the data using the pipeline we described before [33, 34], we performed taxonomic and ARG annotation separately for each sample. For taxonomic annotation, we randomly extracted 1 million reads and aligned them against 16S ribosomal RNA (rRNA) sequences in the SILVA rRNA database (SSURef_132_NR99) [35] using BLASTn with an E-value threshold of 10^− 5^ [36]. We classified the aligned 16S paired-end short reads into bacterial genera using the Ribosomal Database Project (RDP) Classifier [37] and computed the per cent abundance of each genus.

For ARG annotation, we performed de novo assembly of paired-end short reads from each animal into contigs using IDBA-UD [38, 39]. After assembly, we predicted functional genes on contigs using MetaGeneMark [40], mapped short reads to the genes [41], and computed reads per kilobase of transcript per million mapped reads (RPKM) for each gene. We used RPKM as the measure of gene abundance normalized for sequencing depth, gene length, and per-base coverage [42]. Finally, we aligned the predicted genes to the nucleotide sequences in the Comprehensive Antibiotic Resistance Database (CARD) [43] using BLASTn [36]. We determined the genes as ARGs if they were aligned with an E-value threshold of 10^− 5^ and with more than 90% identity and 50% coverage. We obtained the normalized abundance of ARGs by summing the RPKM values of the genes aligned with the same ARG.

### Statistical analysis for the dietary effect on the gut resistome

We analyzed the canine and feline studies separately because their study designs were different. First, we identified the core ARGs, defined as the ARGs present in ≥50% of the samples. Second, we assessed the diversity of ARGs by computing the Shannon diversity index, which accounts for both richness (i.e., the number of different ARGs) and evenness (i.e., the relative abundance of different ARGs) [44]. We hypothesized that an increase in protein and a reduction in carbohydrate in the diet increase gut ARG diversity. To test this hypothesis, we used non-parametric statistical tests because normality could not be assumed in some data. For the canine data, we used the Wilcoxon signed-rank test to compare the diet groups based on samples collected before and after dietary intervention and the Wilcoxon rank-sum test when the comparison was made based only on samples collected after dietary intervention. For the feline data, we used the Wilcoxon rank-sum test. We also computed the Shannon diversity index of bacterial genera and compared between the diet groups with the same statistical tests to assess whether bacterial diversity had the same trend as ARG diversity.

We then assessed whether ARG composition was associated with dietary nutritional content in the following way. We computed Bray-Curtis dissimilarity values for all possible pairs of samples based on the normalized ARG abundance data. Bray-Curtis dissimilarity values range from 0 to 1, with higher values indicating more dissimilar ARG composition between two given samples. Based on these values, we ordinated samples in reduced space using NMDS [45] and performed PERMANOVA tests using the adonis function of the vegan package [46] in R [47] to assess whether the gut microbiota exposed to different dietary nutritional content have different ARG composition [48].

Finally, we performed a Procrustes analysis to test the hypothesis that ARG composition is associated with taxonomic composition in the gut microbiota. Briefly, two NMDS ordinations by ARG and taxonomic composition were uniformly scaled and rotated until the squared differences between them were minimized [49]. We then performed procrustean randomization tests using the protest function of the vegan package (30) in R [47] to assess the correlation between the two NMDS ordinations. For PERMANOVA and procrustean randomization tests, to account for the sampling design, samples were permuted within those collected from the same animals for the canine data and within those collected in the same weeks for feline data.

### Network analysis

**Fig. 7:**
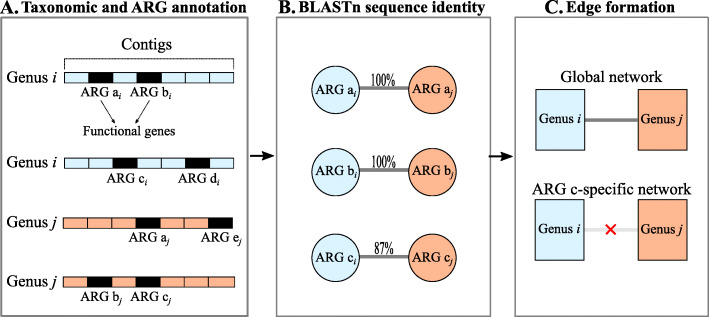
Construction of ARG-sharing networks based on metagenomes. **a** Contigs and their functional genes were annotated as bacterial genus and ARGs, respectively. **b** BLASTn Sequence identity was computed for each pair of functional genes annotated as ARGs. A pair of genes was assumed to represent the same ARG if its BLASTn sequence identity was 100%. **c** In the global network, genera were connected if their contigs shared ≥1 ARG of any type in ≥1 sample among those collected from a given animal species, whereas only the ARG of interest was considered in the ARG-specific network

We constructed networks that described ARG-sharing patterns between gut bacterial genera based on taxonomic and ARG annotation of shotgun metagenomic sequence data (Fig. 7). For taxonomic annotation, we annotated contigs to bacterial genera and phyla using a homology-based taxonomy classifier, MyTaxa [31]. Although MyTaxa has relatively high accuracy at the phylum and genus levels and is considered superior to other annotation tools (30), it was still possible that some contigs were misclassified. Therefore, as a screening step, we considered bacterial genera to be false positives and removed them from the networks if they were determined non-existent in the samples according to 16S rRNA-based taxonomic annotation of short reads. For ARG annotation, we annotated predicted genes to the nucleotide sequences in the CARD [43] using BLASTn. If contigs *C*_*i*_ and *C*_*j*_ annotated to bacterial genera *B*_*i*_ and *B*_*j*_, respectively, contained predicted genes annotated to a specific ARG, *B*_*i*_ and *B*_*j*_ were assumed to share that ARG in their genomes. The predicted genes were assumed to represent the same ARG if their BLASTn sequence identity was 100%, to assess ARG-sharing relationships within the most recent molecular timescale. Networks were constructed for each animal species. They were unweighted and undirected, with nodes representing bacterial genera found to share ARGs in the sampled canine or feline gut microbiota. Two bacterial genera were linked by an edge if at least one ARG was found on contigs belonging to these two genera and originating from the same animal. For each animal species, we constructed two different types of network: (i) global networks including all ARGs identified in the gut microbiota, and (ii) ARG-specific networks for which only one specific ARG was accounted for. For example, while an edge represented the sharing of ≥1 ARG of any kind in the global networks, in a network specific to the tetracycline resistance gene *tet*(W), an edge represented the sharing of ≥1 *tet*(W) genes between two bacterial genera. The global networks showed the overall distribution of ARGs across microbial taxa, whereas ARG-specific networks revealed patterns specific to individual ARGs.

For both network types, we assessed the centrality of each genus by computing the degree, eigenvector, and betweenness using the igraph package [50] in R [47] to identify the most influential genera in the ARG-sharing networks. Degree was the number of other genera with which a given genus shared at least one ARG. Eigenvector accounted for the centrality of the genus and other genera with which it shared at least one ARG [16]. Betweenness quantified the extent to which the genus was laid on paths between other genera [16]. We also examined the degree distribution and correlation between centrality measures using the Kendall rank correlation test in R [47].

The structure of each global network was then characterized. First, we performed a QAP logistic regression to assess whether genera from the same phylum were more likely to share ARGs than with those from different phyla [51, 52]. We used phylum membership as an explanatory variable and ARG sharing as a response variable, and performed the QAP logistic regression using the sna package [53] in R [47]. Second, we identified network communities of genera that shared ARGs more frequently among themselves than with other genera. The fast greedy modularity optimisation algorithm was used to identify the network partition which maximized the modularity (i.e., the extent to which ARG sharing occurs within communities rather than between communities) [16]. We also performed the QAP logistic regression to assess whether genera from the same phylum tended to belong to the same network community, using phylum membership as an explanatory variable and network community membership as a response variable. Finally, we identified groups of genera with similar ARG-sharing patterns by partitioning each network into groups based on structural equivalence. Two genera were considered structurally equivalent if they shared ARGs with the same set of other genera [17]. Ward’s hierarchical clustering method was used to partition each network into groups based on the Euclidian distance between any two genera as the measure of structural equivalence [17, 54, 55]. That is, genera classified as the same group were considered to have similar ARG-sharing patterns.

All *p*-values in this study were adjusted by the false discovery rate [56].

## Supplementary information

**Additional file 1:Table S1.** Summary statistics of de novo assembly.

**Additional file 2:Table S2.** The Kendall rank correlation between centrality metrics in the canine and feline global network.

**Additional file 3:Table S3.** Quadratic Approximation Procedure (QAP) logistic regression results.

**Additional file 4:Table S4.** The edge density within and between structurally equivalent groups in the canine and feline global network.

**Additional file 5: **The ARG-specific network of the canine and feline gut microbiota. (1) ARG-specific network of the canine gut microbiota: **Figure S1** and **Tables S5 and S7.** (2) ARG-specific network of the feline gut microbiota: **Figure S2 and Tables S6 and S8.**

## Data Availability

All shotgun metagenomic sequence datasets are available at the European Nucleotide Archive under the study accession PRJEB20308 (the canine data) and PRJEB4391 (the feline data).

## Abbreviations

ARG: Antibiotic resistance gene
CARD: Comprehensive antibiotic resistance database
HGT: Horizontal gene transfer
HPLC: High-protein and low-carbohydrate diet
LPHC: Low-protein and high-carbohydrate diet
MPMC: Medium-protein and medium-carbohydrate diet
NMDS: Nonmetric multidimensional scaling
PERMANOVA: Permutational multivariate analysis of variance
QAP: Quadratic Approximation Procedure
RPKM: Reads per kilobase of transcript per million mapped reads
