# Supplementary material for: Antibiotic resistance gene sharing networks and the effect of dietary nutritional content on the canine and feline gut resistome
Source: Anim Microbiome. 2020 Feb 7;2:4. doi: 10.1186/s42523-020-0022-2 (PMC7807453; doi:10.1186/s42523-020-0022-2)
Supplement: Supplementary file 1 — Additional file 1:Table S1. Summary statistics of de novo assembly. [file 42523_2020_22_MOESM1_ESM.docx]

| **Table S1. Summary statistics of de novo assembly ^a^** | | |
| --- | --- | --- |
|  | Canine data | Feline data |
| Number of samples ^b^ | 64 | 12 |
| Number of contigs ^c^ | 136,593 (87,095, 186,761) | 120,899 (79,135, 143,975) |
| Number of contigs with ≥ 1 ARG type ^c, d^ | 48.5 (29, 153) | 49 (25, 92) |
| Percent of contigs with ≥ 1 ARG type ^c, d^ | 0.04 (0.02, 0.10) | 0.05 (0.03, 0.08) |
| Number of ARG types ^c, d^ | 32.5 (14, 83) | 34 (19, 79) |
| Number of ARG types shared ^c, d, e^ | 4 (1, 7) | 3.5 (1, 6) |
| a k-mers were iterated from 20 to 120 with a step size of 10 and the minimum contig length of 300 base pairs. Otherwise, default settings were used.  b samples collected from each animal are assembled together.  c median, minimum, and maximum values are reported.  d ARG = antibiotic resistance genes; ARGs are defined as genes aligned to the CARD with < 10^−5^ E-value, > 90% identity, and > 50% coverage.  e the number of ARG types shared between contigs annotated to different bacterial genera. | | |
