# Supplementary material for: Antibiotic resistance gene sharing networks and the effect of dietary nutritional content on the canine and feline gut resistome
Source: Anim Microbiome. 2020 Feb 7;2:4. doi: 10.1186/s42523-020-0022-2 (PMC7807453; doi:10.1186/s42523-020-0022-2)
Supplement: Supplementary file 2 — Additional file 2:Table S2. The Kendall rank correlation between centrality metrics in the canine and feline global network. [file 42523_2020_22_MOESM2_ESM.docx]

| **Table S2. The Kendall rank correlation between centrality metrics in the canine and feline global network** | | | | | |
| --- | --- | --- | --- | --- | --- |
|  | Canine global network | |  | Feline global network | |
|  | Kendall’s tau | p-value |  | Kendall’s tau | p-value |
| Degree vs Betweenness | 0.71 | < 0.001 |  | 0.70 | < 0.001 |
| Degree vs Eigenvector | 0.65 | < 0.001 |  | 0.39 | 0.014 |
| Betweenness vs Eigenvector | 0.45 | < 0.001 |  | 0.31 | 0.057 |
| No. shared ARG types vs Degree | 0.10 | 0.455 |  | -0.19 | 0.293 |
| No. shared ARG types vs Betweenness | 0.12 | 0.354 |  | -0.16 | 0.397 |
| No. shared ARG types vs Eigenvector | -0.04 | 0.719 |  | -0.05 | 0.750 |
| p-values were adjusted by the false discovery rate (FDR) | | | | | |
