# Supplementary material for: Antibiotic resistance gene sharing networks and the effect of dietary nutritional content on the canine and feline gut resistome
Source: Anim Microbiome. 2020 Feb 7;2:4. doi: 10.1186/s42523-020-0022-2 (PMC7807453; doi:10.1186/s42523-020-0022-2)
Supplement: Supplementary file 3 — Additional file 3:Table S3. Quadratic Approximation Procedure (QAP) logistic regression results. [file 42523_2020_22_MOESM3_ESM.docx]

| **Table S3. Quadratic Approximation Procedure (QAP) logistic regression results *^a^*** | | | | | | | | |
| --- | --- | --- | --- | --- | --- | --- | --- | --- |
|  | Community membership *^b^* | | | | Sharing ≥ 1 ARG | | | |
| Taxonomy membership | Canine network | | Feline network | | Canine network | | Feline network | |
|  | OR | p-value | OR | p-value | OR | p-value | OR | p-value |
| Same vs Different *^c^* | 4.59 | < 0.001 | 3.92 | < 0.001 | 4.00 | < 0.001 | 2.25 | 0.164 |
| *a* OR = odds ratio, ARG = antibiotic resistance genes, 5,000 permutations.  *b* community membership determined by the fast greedy modularity optimisation algorithm.  *c* bacterial genera from different phyla were used as the reference category.  p-values were adjusted by the false discovery rate (FDR). | | | | | | | | |
