# Supplementary material for: Antibiotic resistance gene sharing networks and the effect of dietary nutritional content on the canine and feline gut resistome
Source: Anim Microbiome. 2020 Feb 7;2:4. doi: 10.1186/s42523-020-0022-2 (PMC7807453; doi:10.1186/s42523-020-0022-2)
Supplement: Supplementary file 4 — Additional file 4:Table S4. The edge density within and between structurally equivalent groups in the canine and feline global network. [file 42523_2020_22_MOESM4_ESM.docx]

| **Table S4. The edge density within and between structurally equivalent groups in the canine and feline global network** | | | |
| --- | --- | --- | --- |
| Structurally equivalent class | Within central | Within peripheral | Between  central/peripheral |
| Canine global network | | | |
| Edge density (observed network) | 0.78 | 0.05 | 0.11 |
| Edge density (random networks) ^a^ | 0.11 (0, 0.29) | 0.10 (0.07, 0.13) | 0.10 (0.06, 0.15) |
| p-value ^b^ | < 0.001 | < 0.001 | 0.159 |
| Feline global network | | | |
| Edge density (observed network) | 0.76 | 0.06 | 0.14 |
| Edge density (random networks) ^a^ | 0.14 (0, 0.48) | 0.13 (0.07, 0.19) | 0.13 (0.05, 0.20) |
| p-value ^b^ | < 0.001 | < 0.001 | 0.319 |
| a The random networks were generated with the same number of nodes and edges as the observed network using the rgnm function of the sna package in R. The median, the first and the third quartiles values obtained from 5,000 random networks are reported.  b The one-sided p-values are reported. | | | |
