# Supplementary material for: Antibiotic resistance gene sharing networks and the effect of dietary nutritional content on the canine and feline gut resistome
Source: Anim Microbiome. 2020 Feb 7;2:4. doi: 10.1186/s42523-020-0022-2 (PMC7807453; doi:10.1186/s42523-020-0022-2)
Supplement: Supplementary file 5 — Additional file 5: The ARG-specific network of the canine and feline gut microbiota. (1) ARG-specific network of the canine gut microbiota: Figure S1 and Tables S5 and S7. (2) ARG-specific network of the feline gut microbiota: Figure S2 and Tables S6 and S8. [file 42523_2020_22_MOESM5_ESM.docx]

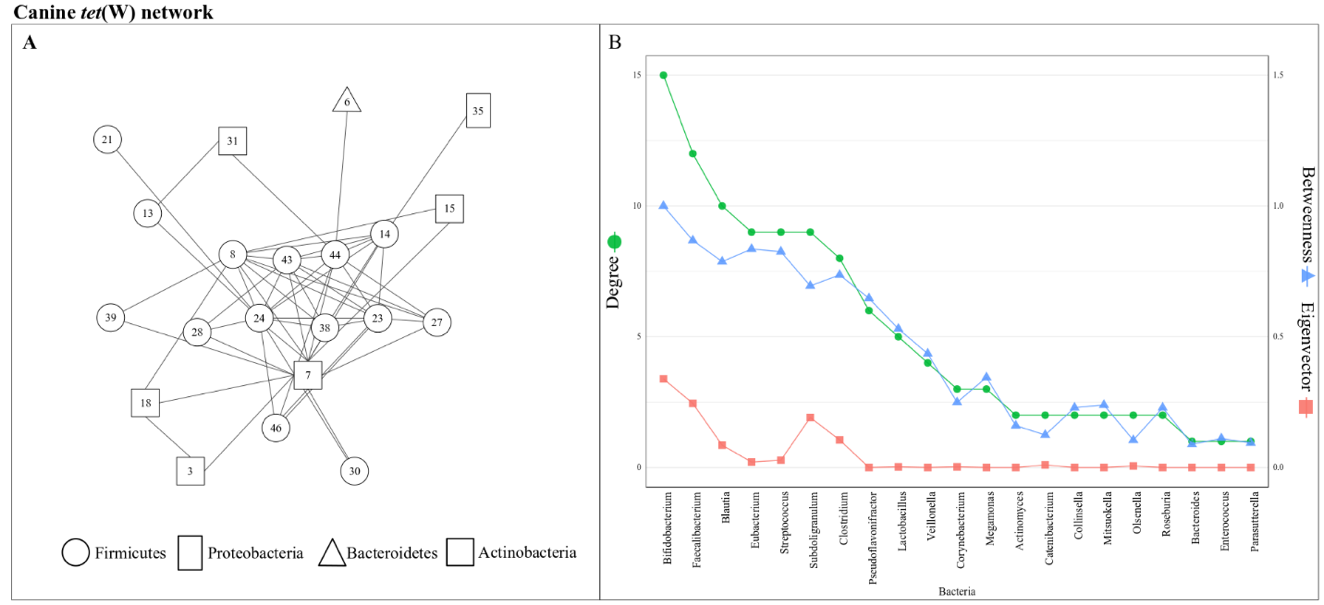


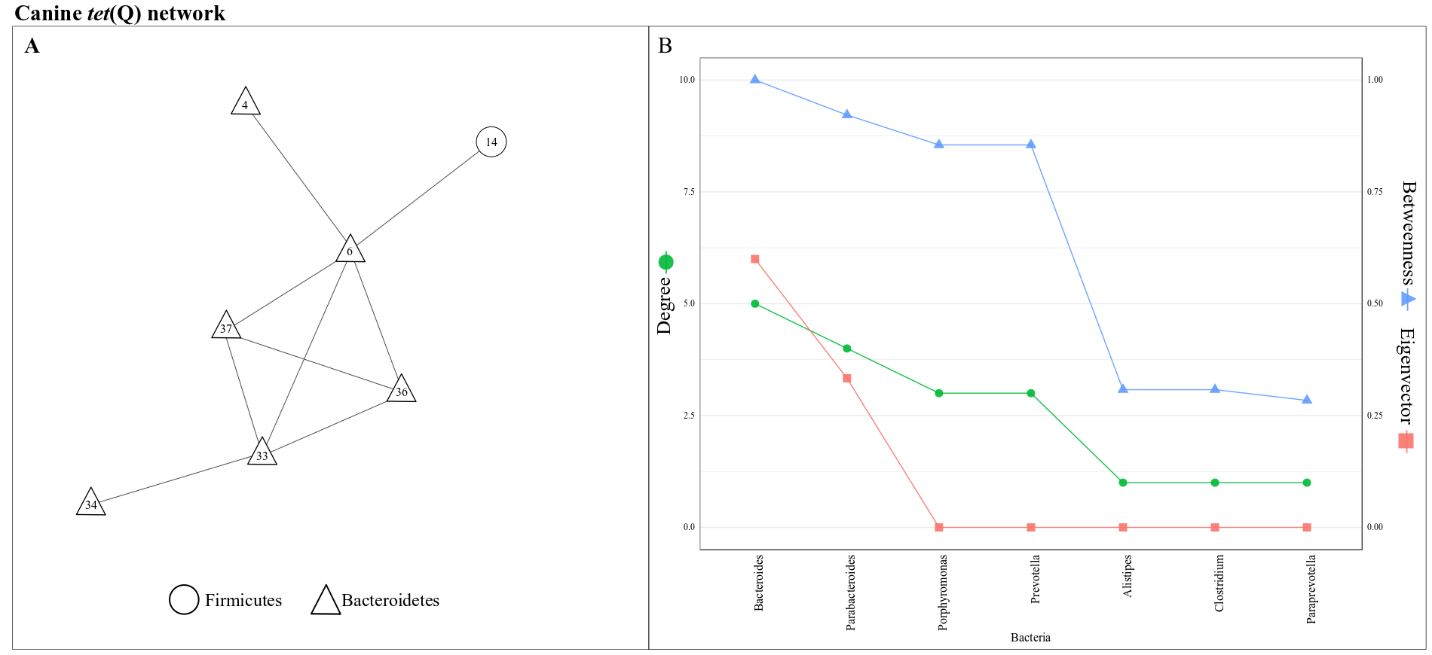


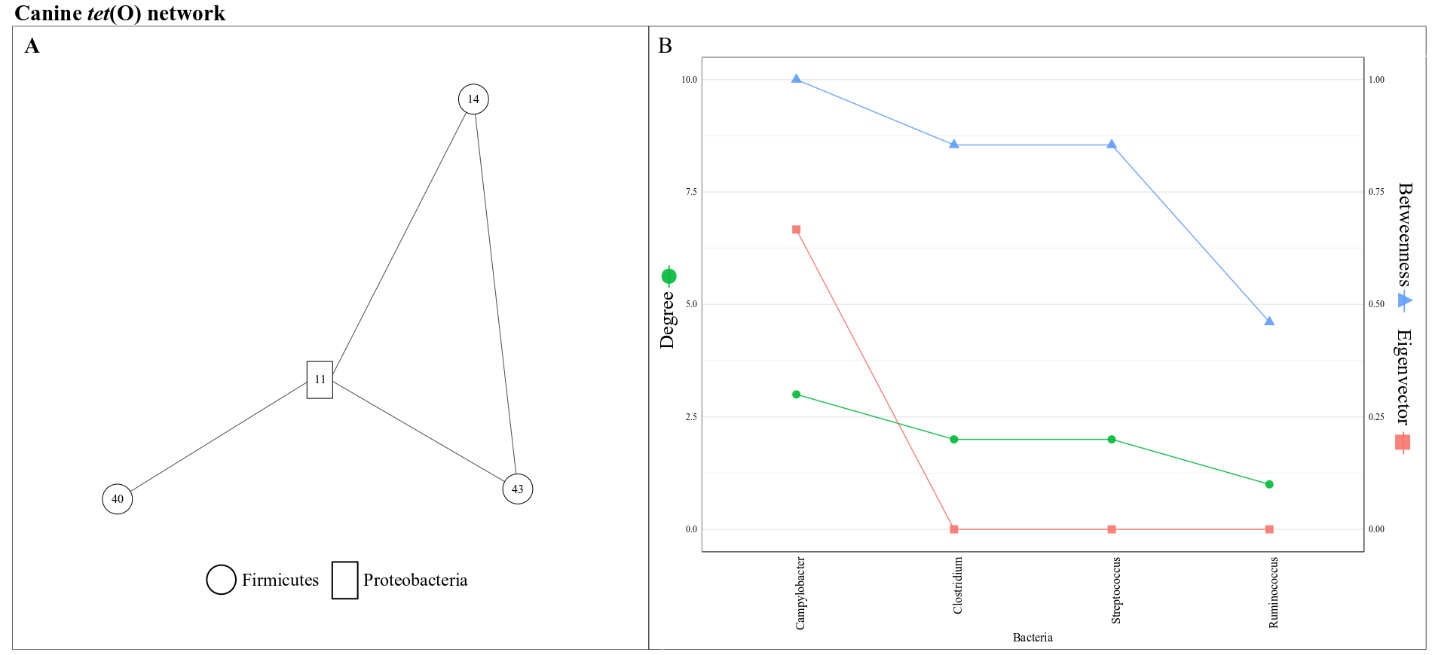


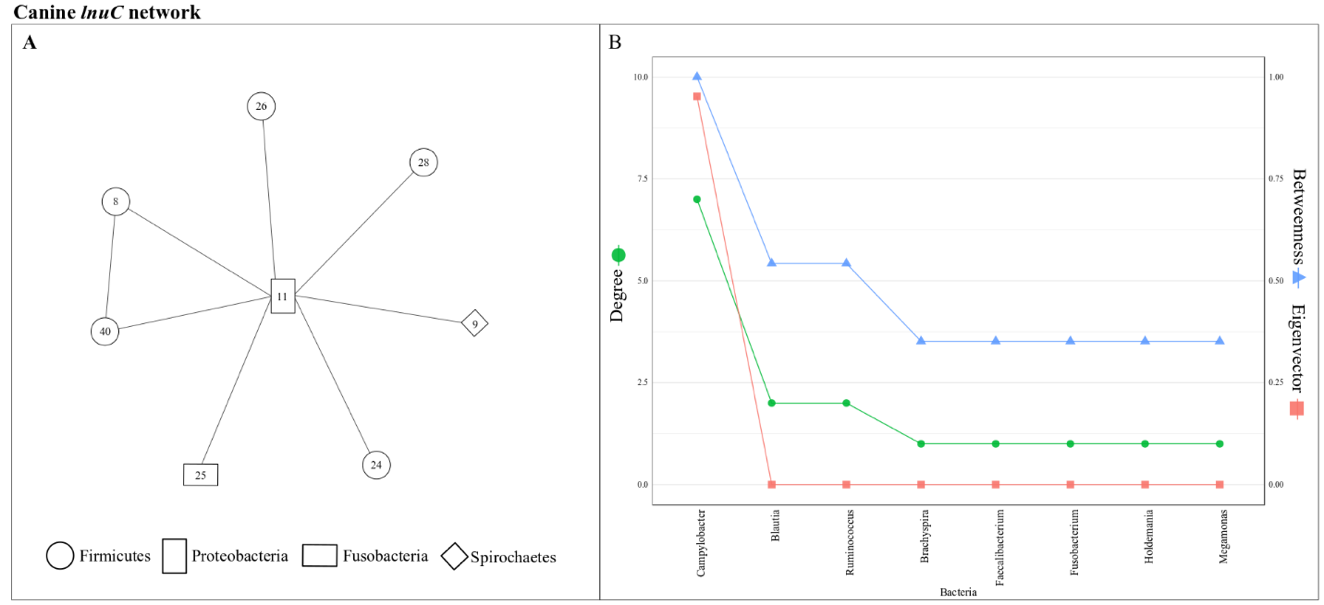


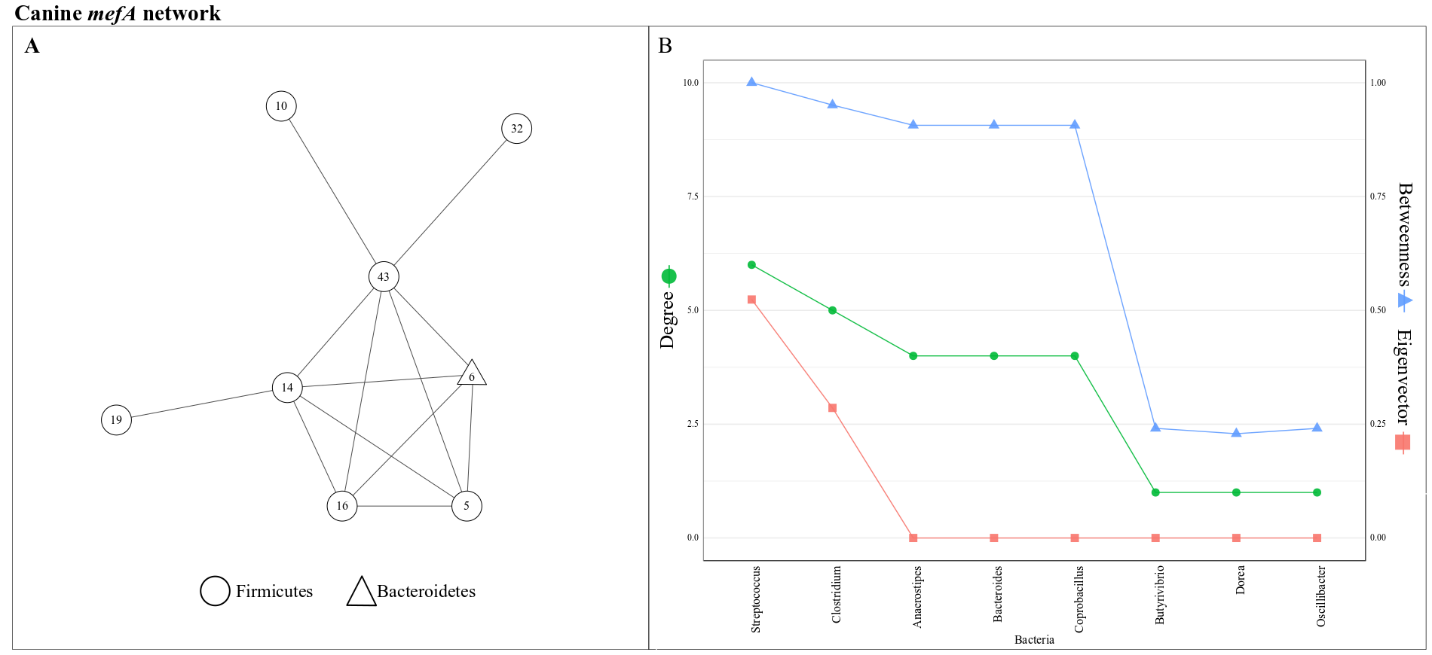


**
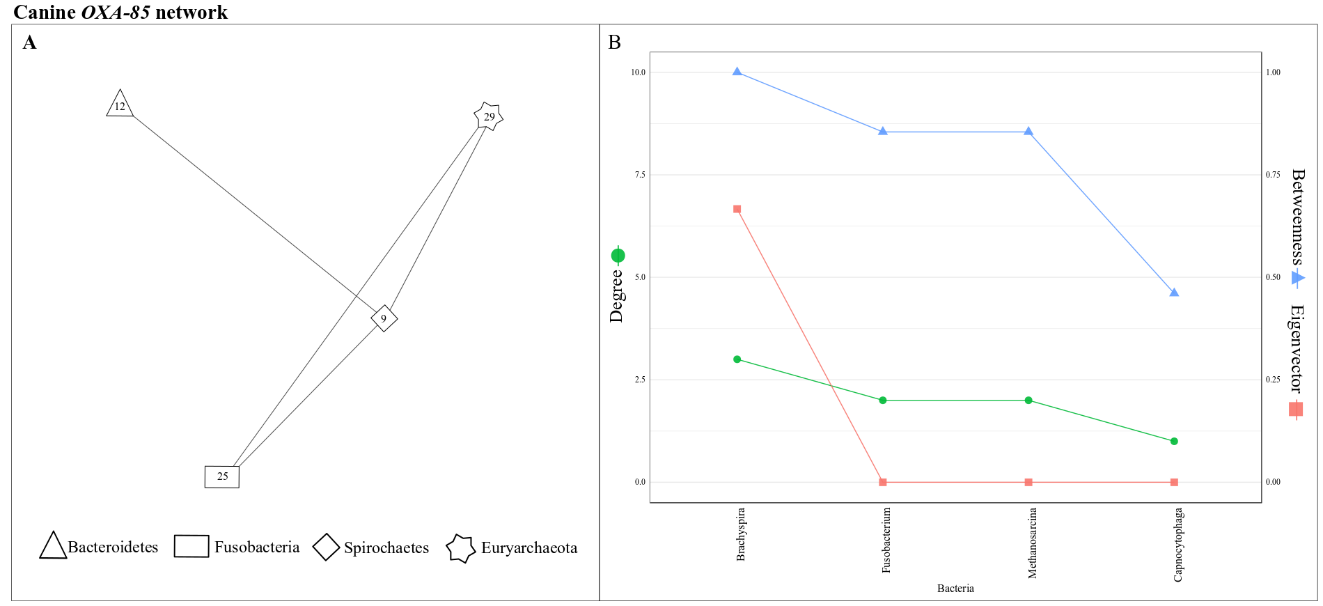
**

**
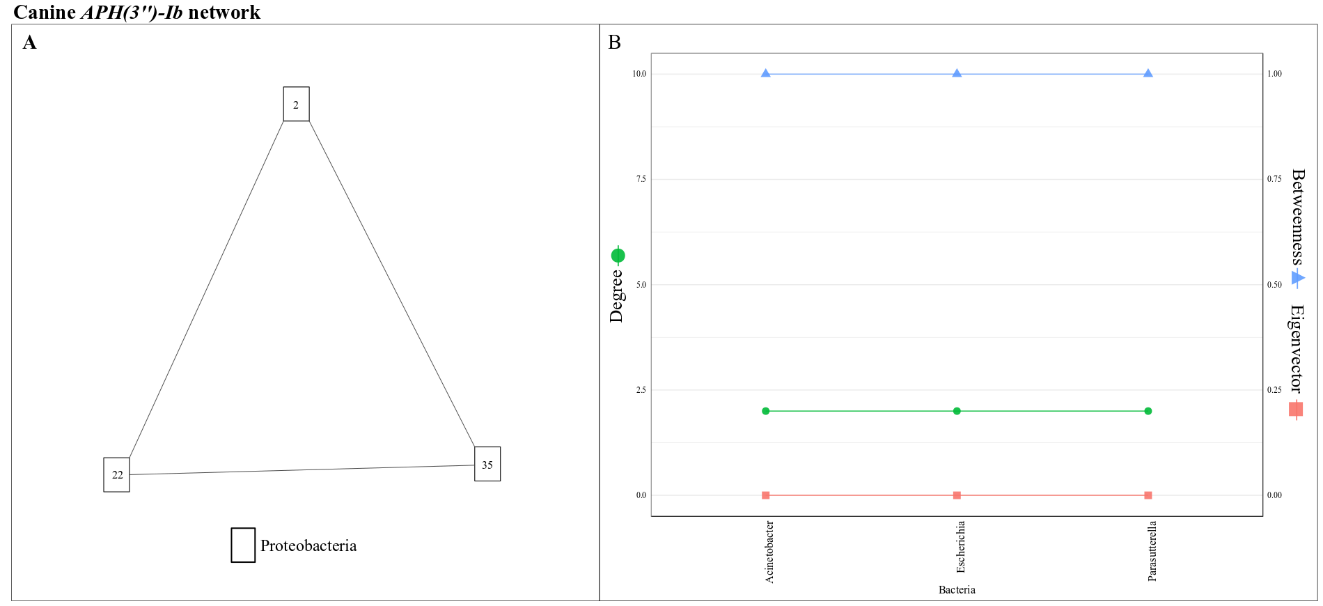
**

**
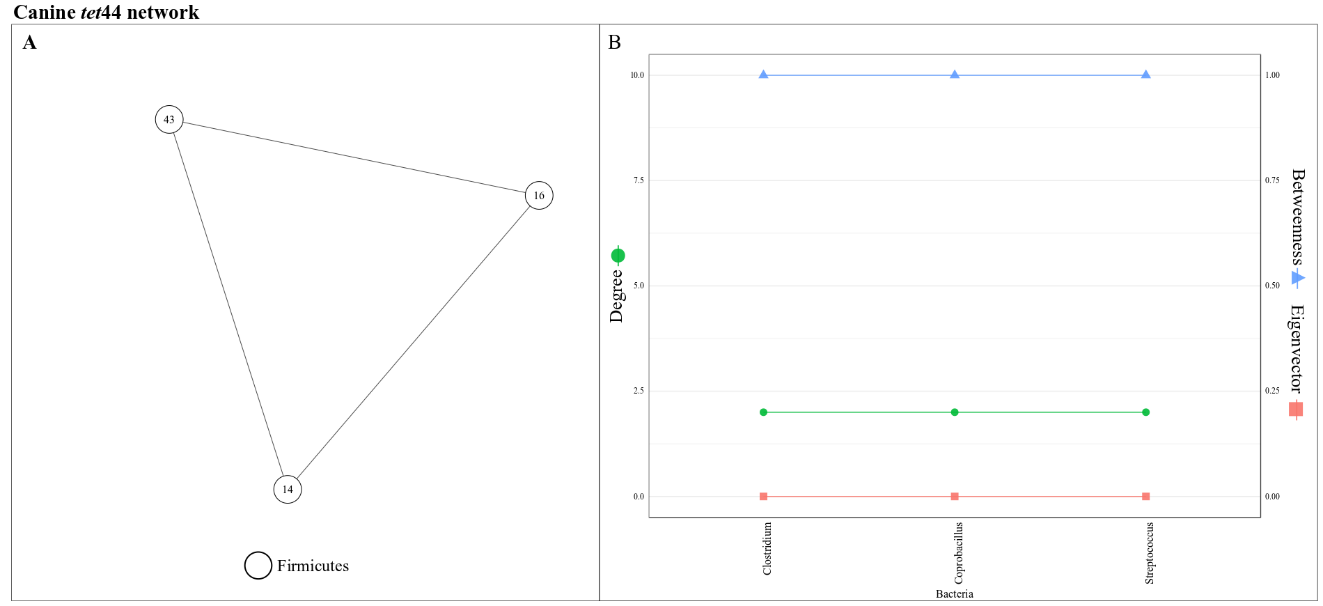
**

**
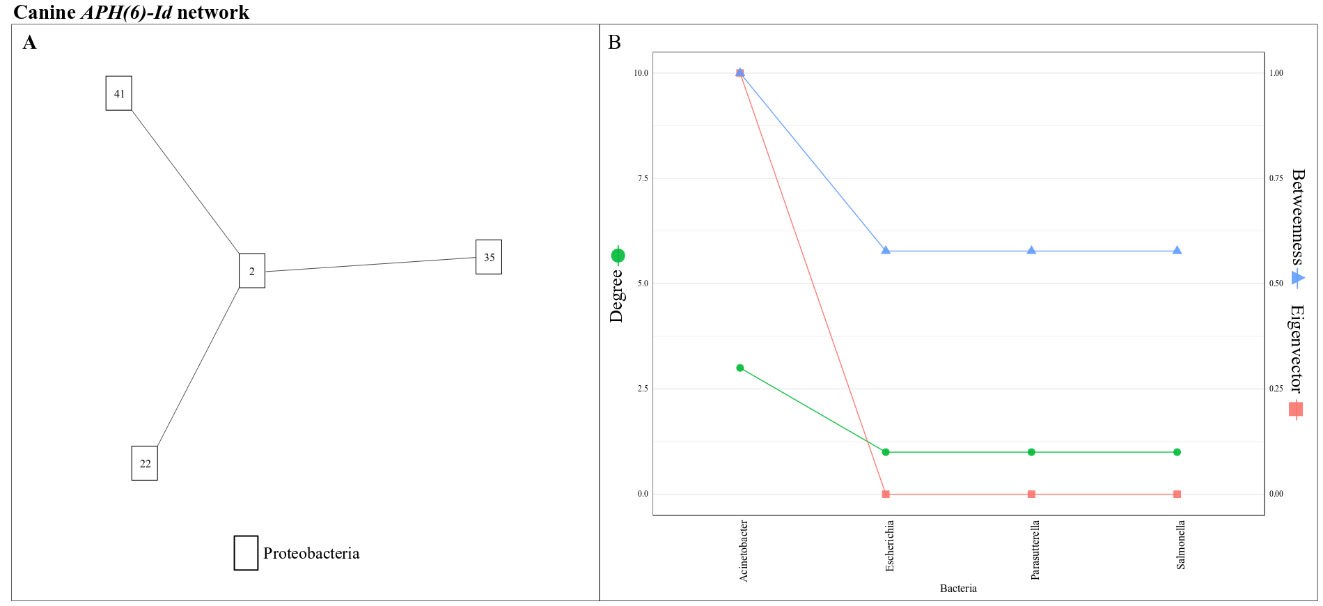
**

**
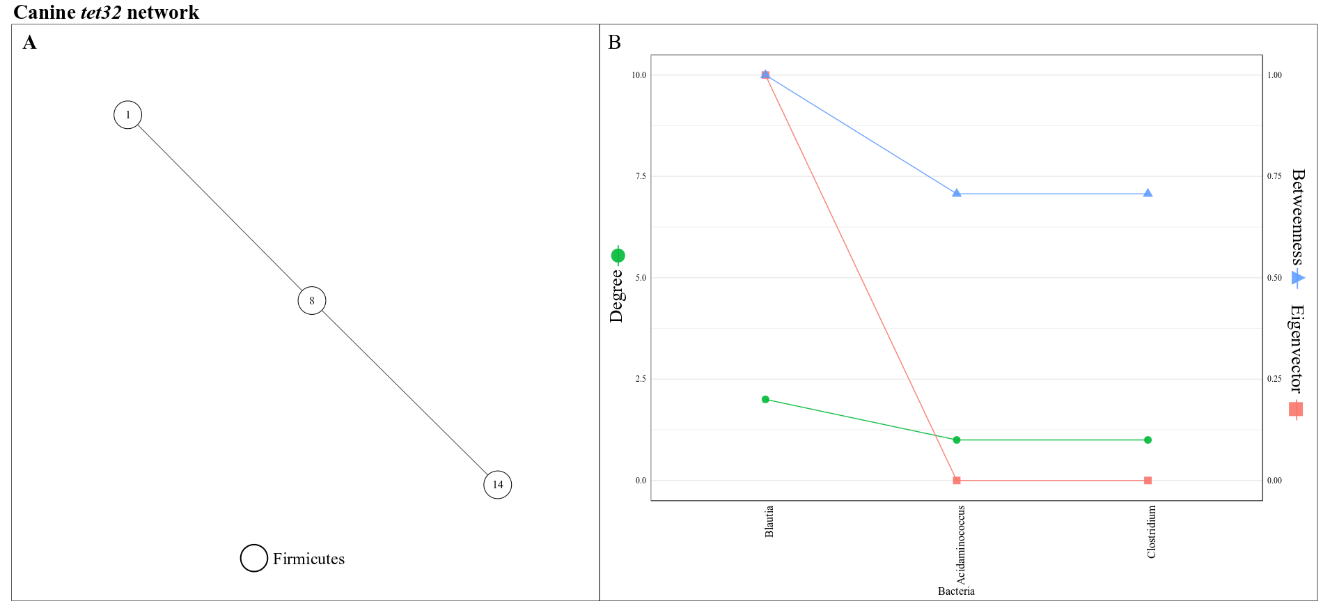
**

**
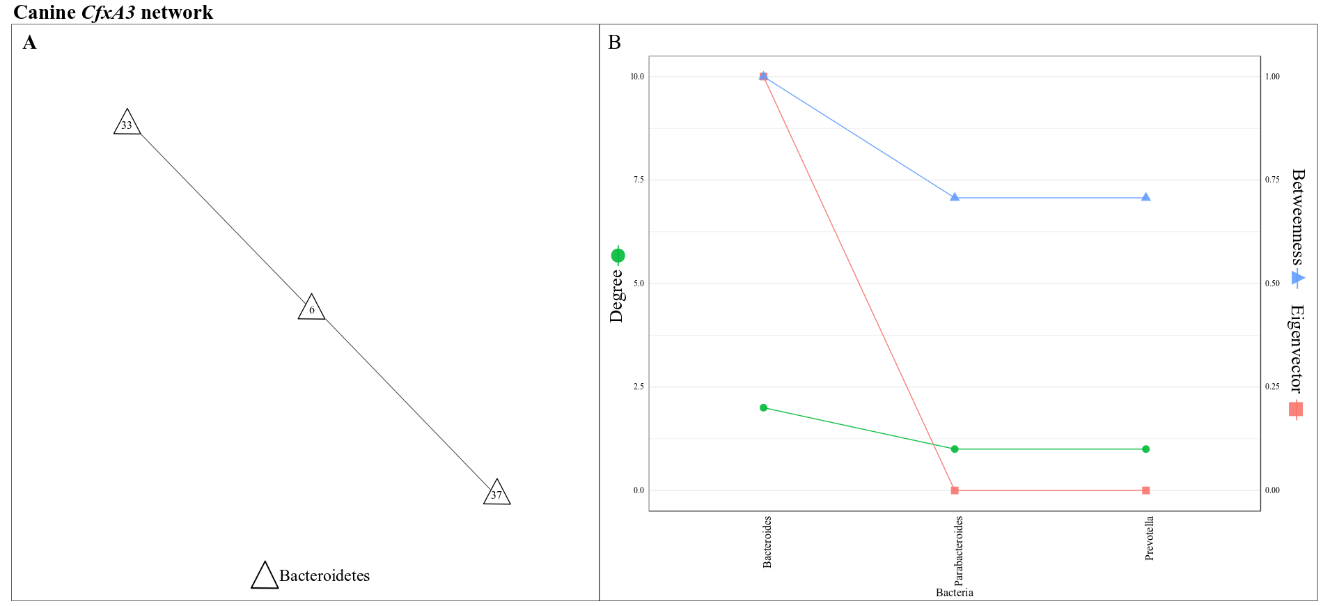
**

**
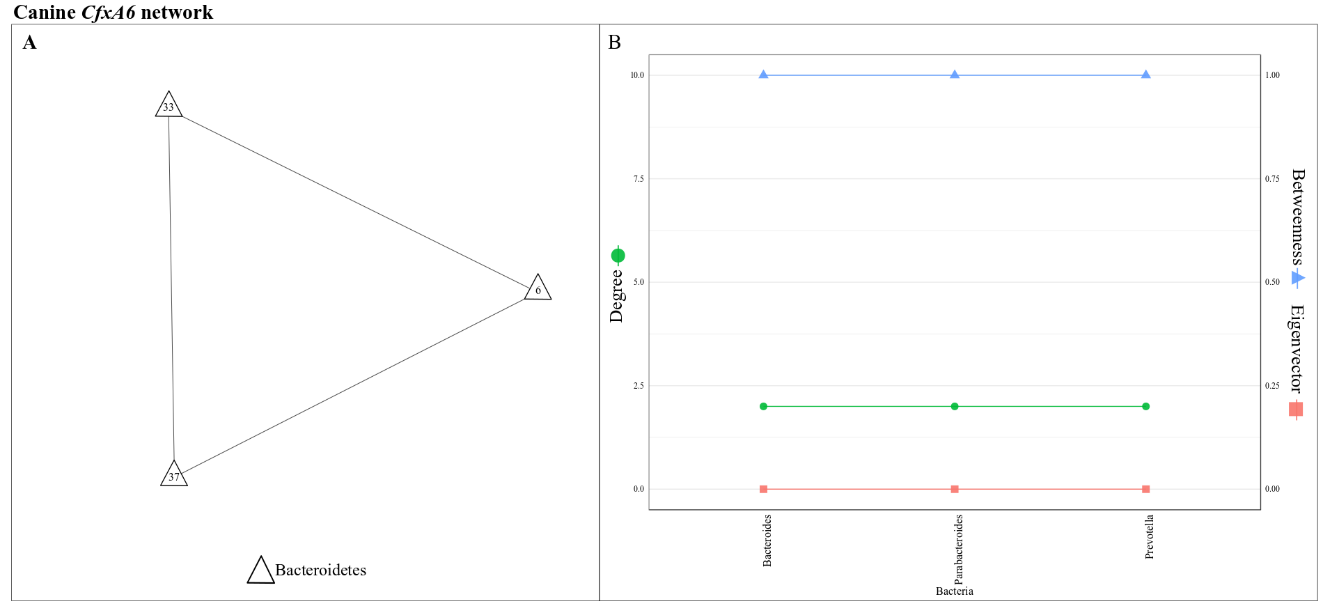
**

**Figure S1.** The ARG-specific network of the canine gut microbiota. **(A)** represents the network graph for a given ARG found in ≥ 50% of the samples. Nodes represented genera, with their shapes and colors representing phylum and community memberships, respectively. Two genera were connected by an edge if their contigs shared a given ARG in ≥ 1 sample. Node labels are IDs of genera (Suppl. Table 3). **(B)** represents centrality in the corresponding ARG-specific network. Only the ARGs shared by more than three bacterial genera are presented (See Suppl. Table 5 for those shared only by two genera).

| **Table S5.** Antibiotic resistance genes (ARGs) shared only by two bacterial genera in the canine gut microbiota | | | | |
| --- | --- | --- | --- | --- |
| ARG | Bacteria 1 | | Bacteria 2 | |
|  | Genus | Phylum | Genus | Phylum |
| tetM | Enterococcus | Firmicutes | Streptococcus | Firmicutes |
| bacA | Escherichia | Proteobacteria | Salmonella | Proteobacteria |
| lnuA | Enterococcus | Firmicutes | Staphylococcus | Firmicutes |
| mdtM | Enterobacter | Proteobacteria | Escherichia | Proteobacteria |
| aadA2 | Acinetobacter | Proteobacteria | Escherichia | Proteobacteria |
| mdtO | Escherichia | Proteobacteria | Salmonella | Proteobacteria |
| cmeB | Campylobacter | Proteobacteria | Turicibacter | Firmicutes |
| AAC(6’)-Ip | Bifidobacterium | Actinobacteria | Clostridium | Firmicutes |
| mdtP | Escherichia | Proteobacteria | Salmonella | Proteobacteria |


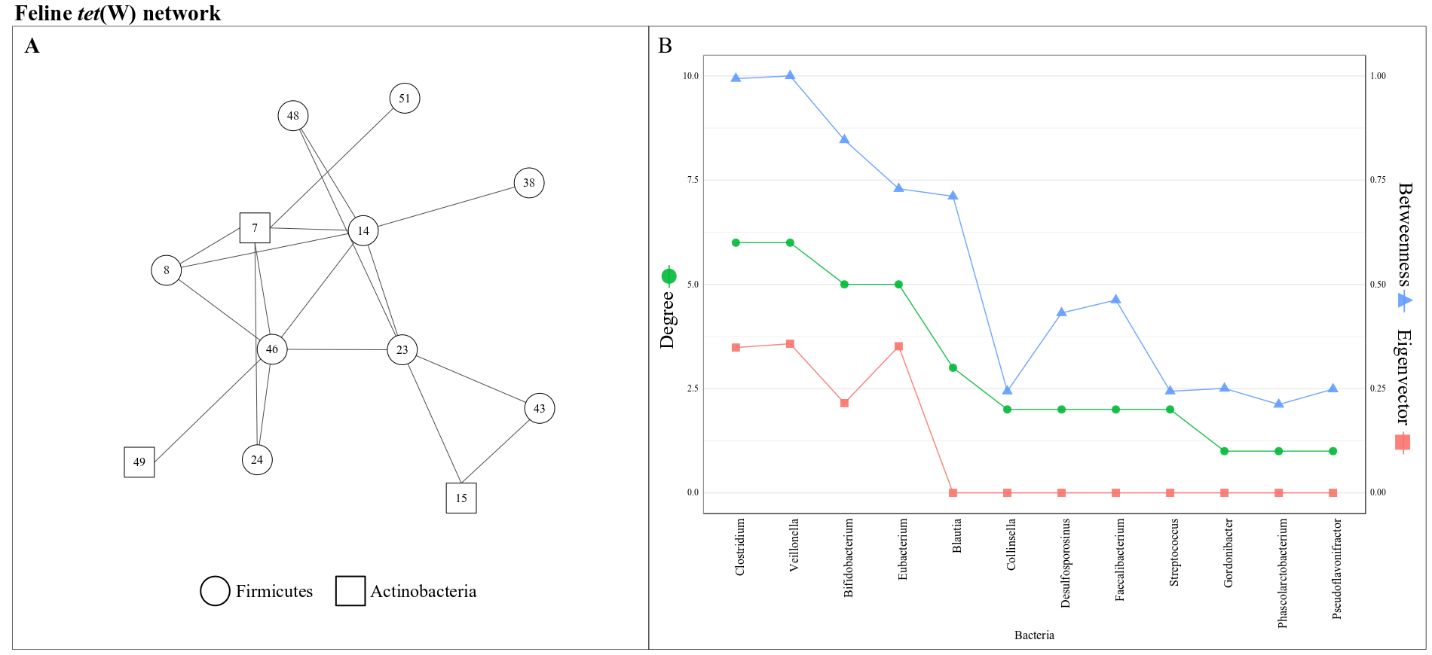


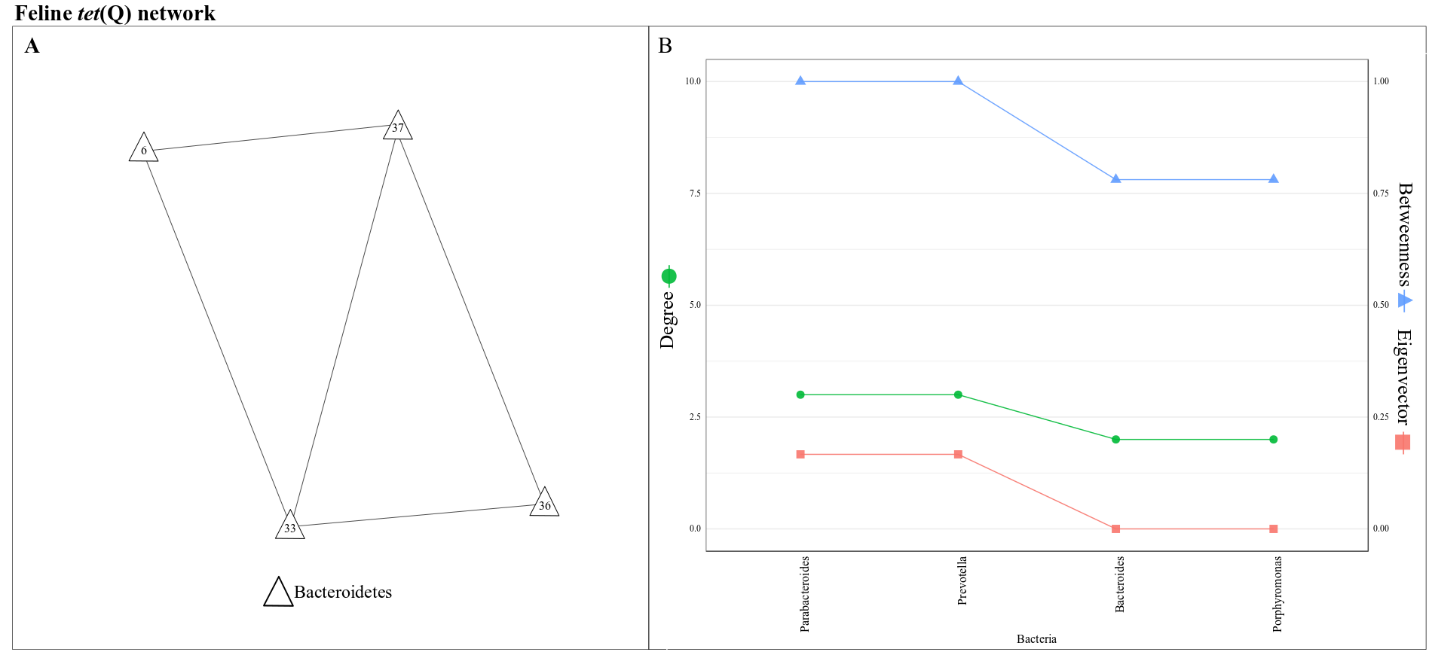


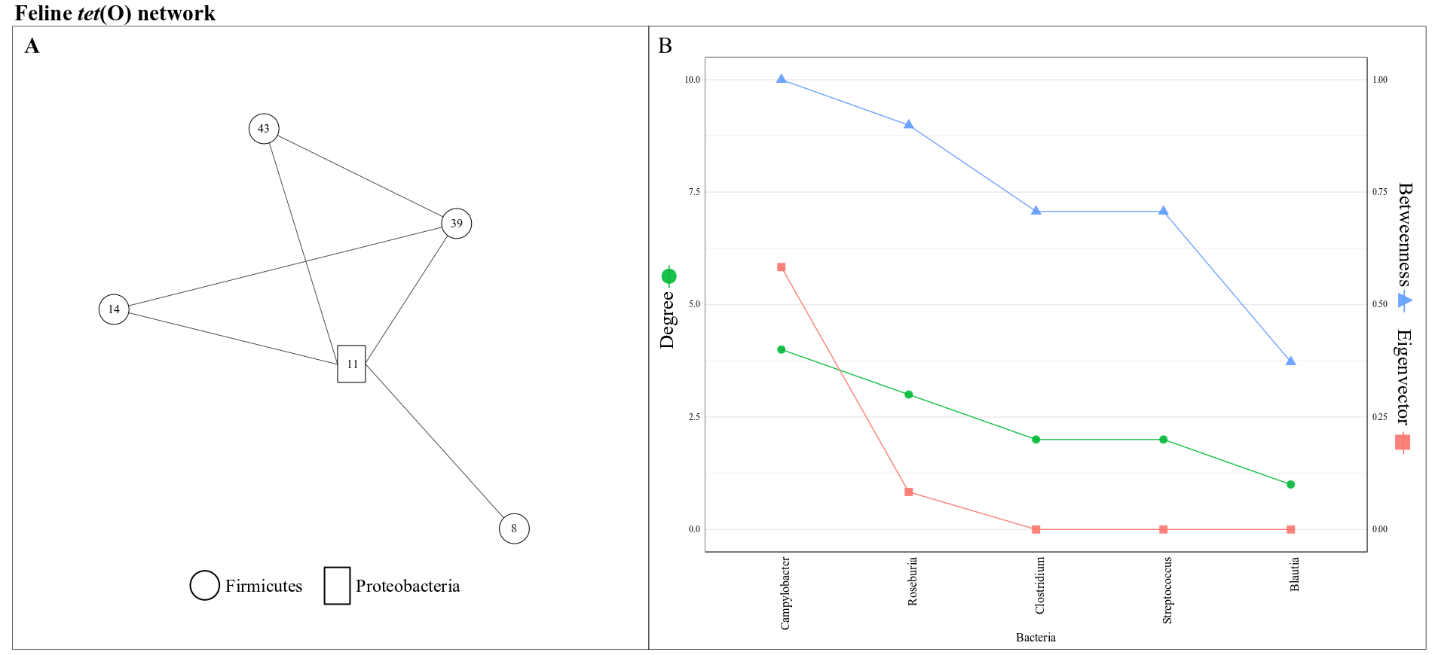


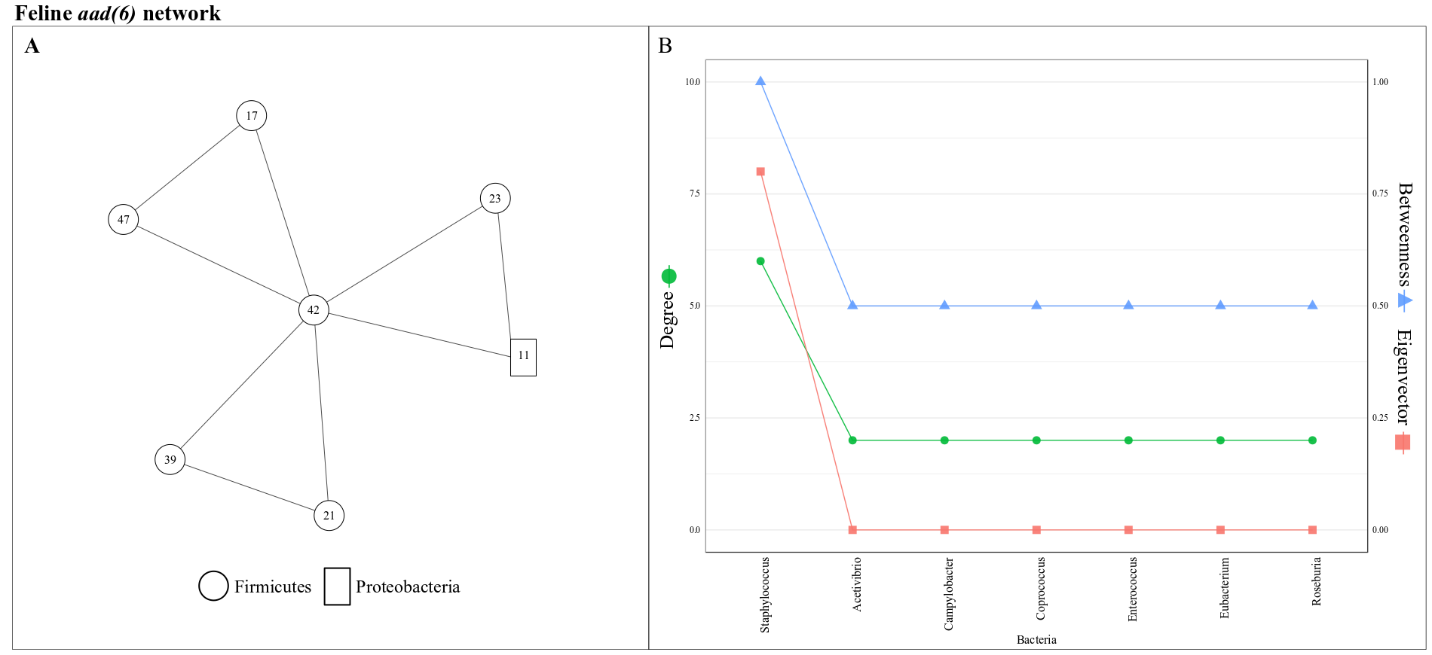


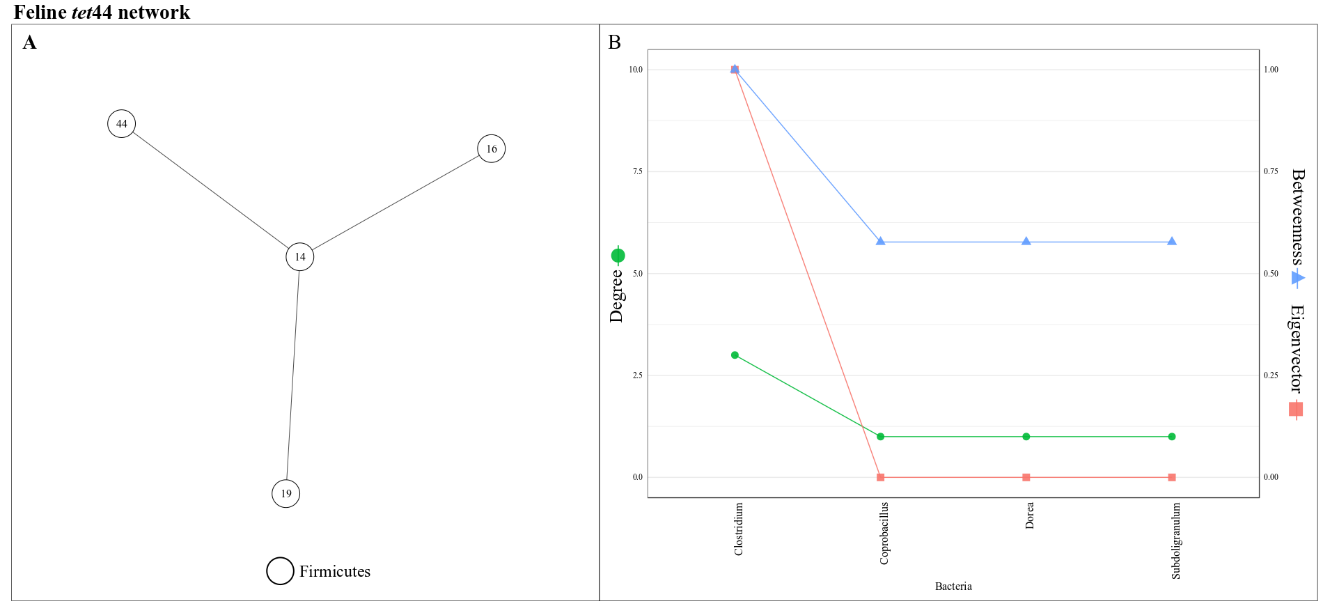


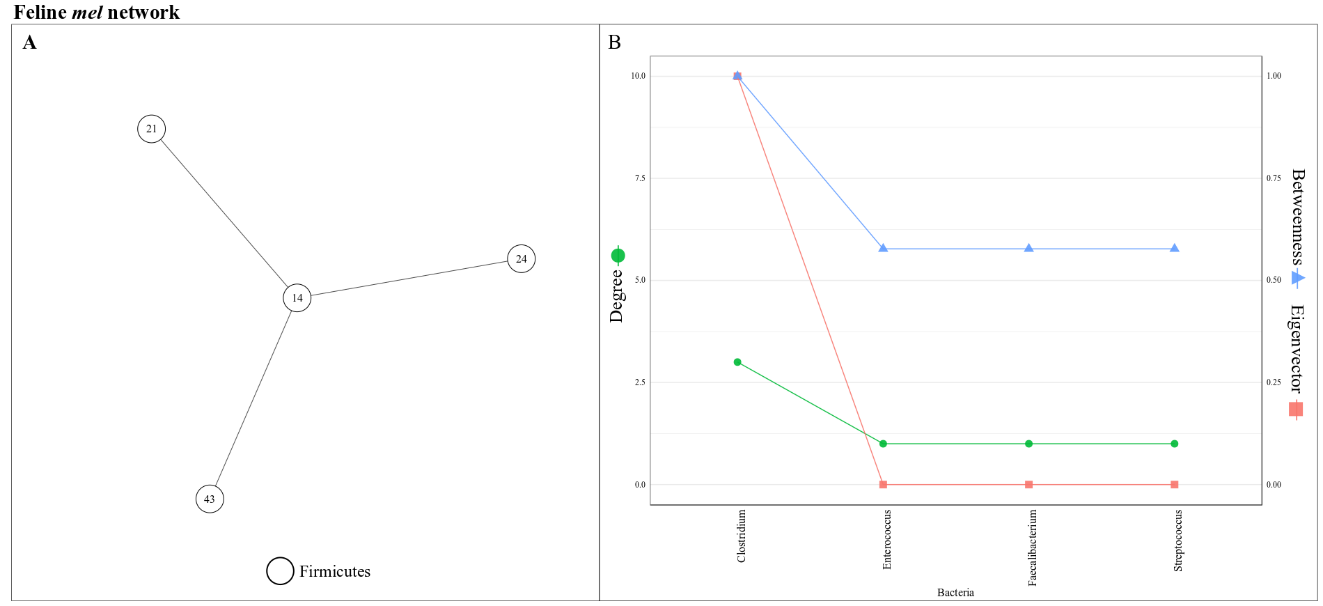


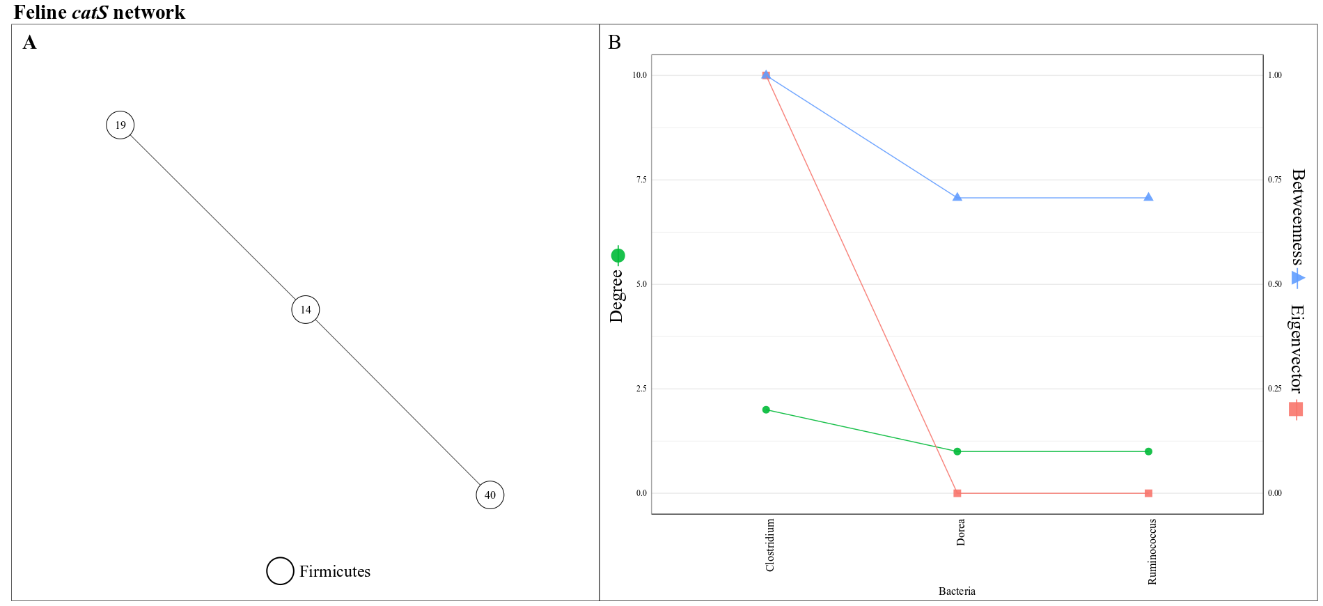


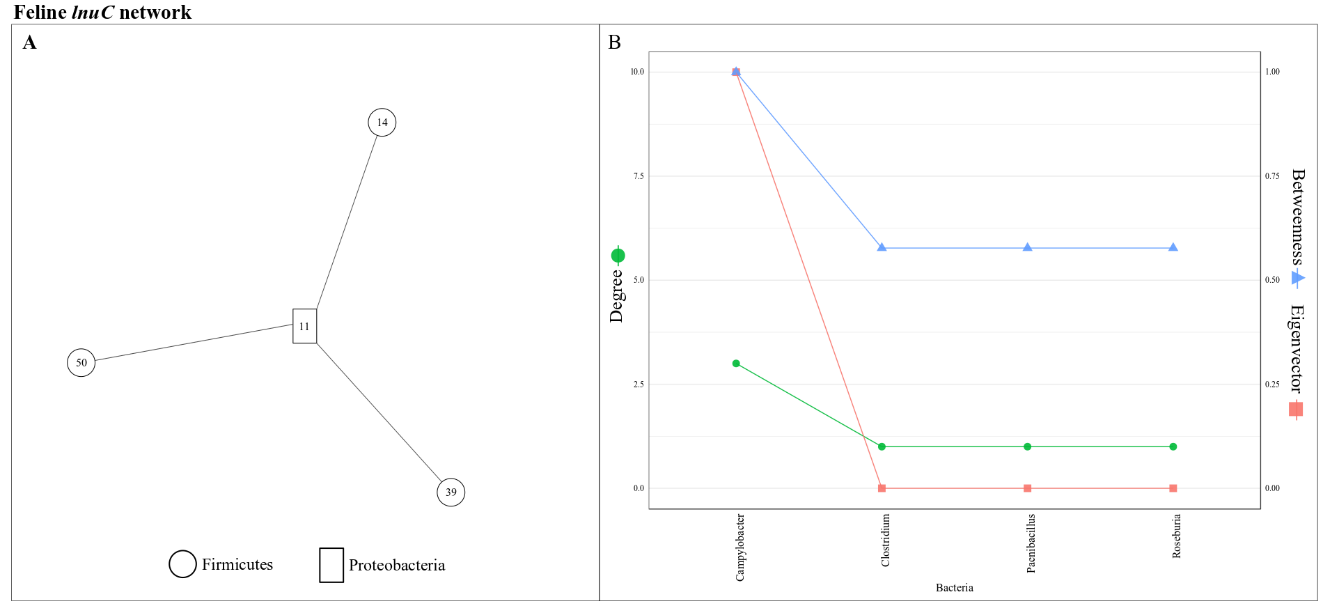


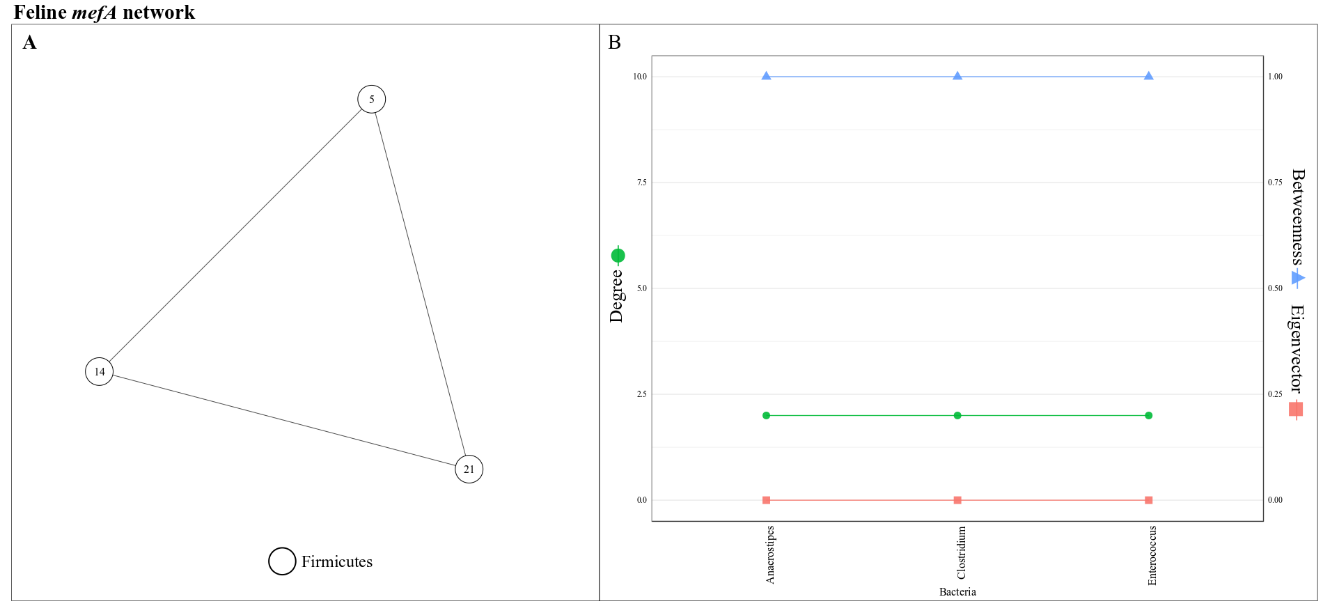


**Figure S2.** The ARG-specific network of the feline gut microbiota. **(A)** represents the network graph for a given ARG found in ≥ 50% of the samples. Nodes represented genera, with their shapes and colors representing phylum and community memberships, respectively. Two genera were connected by an edge if their contigs shared a given ARG in ≥ 1 sample. Node labels are IDs of genera (Suppl. Table 3). **(B)** represents centrality in the corresponding ARG-specific network. Only the ARGs shared by more than three bacterial genera are presented (See Suppl. Table 6 for those shared only by two genera).

| **Table S6.** Antibiotic resistance genes (ARGs) shared only by two bacterial genera in the feline gut microbiota | | | | |
| --- | --- | --- | --- | --- |
| ARG | Bacteria 1 | | Bacteria 2 | |
|  | Genus | Phylum | Genus | Phylum |
| ErmB | Enterococcus | Firmicutes | Streptococcus | Firmicutes |
| SAT-4 | Enterococcus | Firmicutes | Staphylococcus | Firmicutes |

| **Table S7. The number of genera by phylum membership in the canine ARG-specific networks** | | | | | | | | |
| --- | --- | --- | --- | --- | --- | --- | --- | --- |
| ARG type | Phylum | | | | | | | Total ^a^ |
|  | Firmicutes | Bacteroidetes | Actinobacteria | Spirochaetes | Proteobacteria | Fusobacteria | Euryarchaeota |  |
| tetW | 14 | 1 | 5 | 0 | 1 | 0 | 0 | 21 |
| mefA | 7 | 1 | 0 | 0 | 0 | 0 | 0 | 8 |
| tetO | 3 | 0 | 0 | 0 | 1 | 0 | 0 | 4 |
| tetQ | 1 | 6 | 0 | 0 | 0 | 0 | 0 | 7 |
| mel | 7 | 1 | 0 | 0 | 0 | 1 | 0 | 9 |
| lnuC | 5 | 0 | 0 | 1 | 1 | 1 | 0 | 8 |
| OXA-85 | 0 | 1 | 0 | 1 | 0 | 1 | 1 | 4 |
| APH(3'')-Ib | 0 | 0 | 0 | 0 | 3 | 0 | 0 | 3 |
| tetM | 2 | 0 | 0 | 0 | 0 | 0 | 0 | 2 |
| APH(6)-Id | 0 | 0 | 0 | 0 | 4 | 0 | 0 | 4 |
| tet44 | 3 | 0 | 0 | 0 | 0 | 0 | 0 | 3 |
| CfxA3 | 0 | 3 | 0 | 0 | 0 | 0 | 0 | 3 |
| CfxA6 | 0 | 3 | 0 | 0 | 0 | 0 | 0 | 3 |
| tet32 | 3 | 0 | 0 | 0 | 0 | 0 | 0 | 3 |
| mdtM | 0 | 0 | 0 | 0 | 2 | 0 | 0 | 2 |
| lnuA | 2 | 0 | 0 | 0 | 0 | 0 | 0 | 2 |
| bacA | 0 | 0 | 0 | 0 | 2 | 0 | 0 | 2 |
| cmeB | 1 | 0 | 0 | 0 | 1 | 0 | 0 | 2 |
| AAC(6')-Ip | 1 | 0 | 1 | 0 | 0 | 0 | 0 | 2 |
| aadA2 | 0 | 0 | 0 | 0 | 2 | 0 | 0 | 2 |
| mdtO | 0 | 0 | 0 | 0 | 2 | 0 | 0 | 2 |
| mdtP | 0 | 0 | 0 | 0 | 2 | 0 | 0 | 2 |
| a The number of genera in each canine ARG-specific network | | | | | | | | |

| **Table S8. The number of genera by phylum membership in the feline ARG-specific networks** | | | | | |
| --- | --- | --- | --- | --- | --- |
| ARG type | Phylum | | | | Total ^a^ |
|  | Firmicutes | Bacteroidetes | Actinobacteria | Proteobacteria |  |
| tetW | 9 | 0 | 3 | 0 | 12 |
| tetQ | 0 | 4 | 0 | 0 | 4 |
| tetO | 4 | 0 | 0 | 1 | 5 |
| tet44 | 4 | 0 | 0 | 0 | 4 |
| Mel | 4 | 0 | 0 | 0 | 4 |
| Aad(6) | 6 | 0 | 0 | 1 | 7 |
| ErmB | 2 | 0 | 0 | 0 | 2 |
| catS | 3 | 0 | 0 | 0 | 3 |
| lnuC | 3 | 0 | 0 | 1 | 4 |
| mefA | 3 | 0 | 0 | 0 | 3 |
| SAT-4 | 2 | 0 | 0 | 0 | 2 |
| a The number of genera in each feline ARG-specific network | | | | | |
